# *De novo* Assembly of the Pokeweed Genome Provides Insight Into Pokeweed Antiviral Protein (PAP) Gene Expression

**DOI:** 10.3389/fpls.2019.01002

**Published:** 2019-08-06

**Authors:** Kira C. M. Neller, Camille A. Diaz, Adrian E. Platts, Katalin A. Hudak

**Affiliations:** ^1^Department of Biology, York University, Toronto, ON, Canada; ^2^Department of Biology, Center for Genomics and Systems Biology, New York University, New York, NY, United States

**Keywords:** genome assembly, *cis* regulatory element, intron mediated enhancement, jasmonic acid, *Phytolacca americana*, pokeweed, pokeweed antiviral protein, ribosome inactivating protein

## Abstract

Ribosome-inactivating proteins (RIPs) are RNA glycosidases thought to function in defense against pathogens. These enzymes remove purine bases from RNAs, including rRNA; the latter activity decreases protein synthesis *in vitro*, which is hypothesized to limit pathogen proliferation by causing host cell death. Pokeweed antiviral protein (PAP) is a RIP synthesized by the American pokeweed plant (*Phytolacca americana*). PAP inhibits virus infection when expressed in crop plants, yet little is known about the function of PAP in pokeweed due to a lack of genomic tools for this non-model species. In this work, we *de novo* assembled the pokeweed genome and annotated protein-coding genes. Sequencing comprised paired-end reads from a short-insert library of 83X coverage, and our draft assembly (N50 = 42.5 Kb) accounted for 74% of the measured pokeweed genome size of 1.3 Gb. We obtained 29,773 genes, 73% of which contained known protein domains, and identified several PAP isoforms. Within the gene models of each PAP isoform, a long 5′ UTR intron was discovered, which was validated by RT-PCR and sequencing. Presence of the intron stimulated reporter gene expression in tobacco. To gain further understanding of PAP regulation, we complemented this genomic resource with expression profiles of pokeweed plants subjected to stress treatments [jasmonic acid (JA), salicylic acid, polyethylene glycol, and wounding]. Cluster analysis of the top differentially expressed genes indicated that some PAP isoforms shared expression patterns with genes involved in terpenoid biosynthesis, JA-mediated signaling, and metabolism of amino acids and carbohydrates. The newly sequenced promoters of all PAP isoforms contained *cis*-regulatory elements associated with diverse biotic and abiotic stresses. These elements mediated response to JA in tobacco, based on reporter constructs containing promoter truncations of PAP-I, the most abundant isoform. Taken together, this first genomic resource for the Phytolaccaceae plant family provides new insight into the regulation and function of PAP in pokeweed.

## Introduction

American pokeweed, *Phytolacca americana*, belongs to the Phytolaccaceae family of flowering plants, which comprises 65 species of herbs, shrubs, and trees. *P. americana* (pokeweed) is the most well-studied *Phytolacca* species due to its broad agricultural and medical applications. Pokeweed synthesizes PAP, an *N*-glycosidase and RIP that depurinates the conserved α-sarcin loop of large rRNAs (Endo et al., 1988). PAP exhibits antiviral activity against diverse plant and animal viruses. Specifically, depurination of rRNA inactivates ribosomes in infected cells, thereby inhibiting host and viral protein synthesis (Lodge et al., 1993; Bonness et al., 1994). PAP also depurinates the genomes of some RNA viruses, interfering with multiple stages of the viral life cycle (He et al., 2008; Karran and Hudak, 2008; Mansouri et al., 2009). Transgenic plants expressing PAP acquire novel antiviral and antifungal activities, making the gene an attractive candidate for use in agricultural engineering (Zoubenko et al., 1997, 2000; Wang et al., 1998; Dai et al., 2003). Pokeweed also shows potential in phytoremediation as a heavy metal hyperaccumulator, thriving in contaminated soil that is otherwise toxic to most plants (Peng et al., 2008; Liu et al., 2010; Zhao et al., 2011). Although this non-model plant displays resistance to diverse biotic and abiotic stresses, a wealth of information remains unknown since its genome has not been sequenced.

Several isoforms of PAP have been reported, exhibiting different temporal (PAP-I, PAP-II, PAP-III) or tissue-specific (PAP-R, PAP-S, S1, S2, PAP-α) expression patterns, or identified during cell culture (PAP-H, PAP-C) (Irvin, 1975; Irvin et al., 1980; Barbieri et al., 1982, 1989; Bolognesi et al., 1990; Kataoka et al., 1992; Rajamohan et al., 1999; Park et al., 2002). Consistent with a hypothesized role in pathogen defense, we showed previously through transcriptomic analysis that expression of several PAP isoforms is up-regulated by JA (Neller et al., 2016). JA is a plant hormone that mediates resistance to insect herbivores, which are viral vectors, and necrotrophic pathogens. Others have reported an induction of RIP expression in various plants upon treatment with phytohormones [JA, salicylic acid (SA), abscisic acid (ABA)] or associated stresses, including insect feeding, pathogen infection, cold, heat, drought, salinity, and mechanical wounding (Reinbothe et al., 1994; Song et al., 2000; Iglesias et al., 2005; Jiang et al., 2008; Qin et al., 2009; Tartarini et al., 2010). Therefore, it is well-established that RIPs are induced by various stresses; however, it is not clear how RIP isoform expression is controlled, or how RIPs are integrated within stress-response pathways.

Pokeweed is tetraploid with a chromosome count of 2n = 36 and 1C-value of 1.48 pg (∼1.5 Gb) (Bennett, 2000; Rice et al., 2015). Although there are no reference genomes available for the Phytolaccaceae family, genomes of some members from the order Caryophylalles (which includes pokeweed) have been sequenced. Genome assembly and annotation has been performed for *Beta vulgaris* (sugar beet), *Spinacia oleracea* (spinach), *Chenopodium quinoa* (quinoa), and *Amaranthus hypochondriacus* (amaranth) (Dohm et al., 2014; Clouse et al., 2016; Jarvis et al., 2017; Xu et al., 2017). This genomic information is a useful reference for pokeweed, especially since RIPs are prevalent among the Caryophyllales (Di Maro et al., 2014).

Here, we present the first *de novo* draft genome assembly of pokeweed and an annotation of its protein-coding genes. Using this resource, we investigated the presence and gene organization of PAP isoforms. A novel feature was discovered in the 5′ UTR: a long intron that affects gene expression. Integration of RNA-Seq data from pokeweed stress treatments enabled the identification of co-expressed genes and provided insight into defense responses in the plant. Finally, differences in CREs in the promoters of PAP isoforms, combined with their unique expression profiles, suggest that the isoforms have distinct roles in pokeweed. Our study provides a workflow that integrates genome assembly, annotation, and differential expression analysis for a non-model plant. These resources will facilitate the study of pokeweed to understand its ability to survive environmental stress.

## Materials and Methods

### Genomic DNA Sequencing and Genome Assembly

**FIGURE 1:**
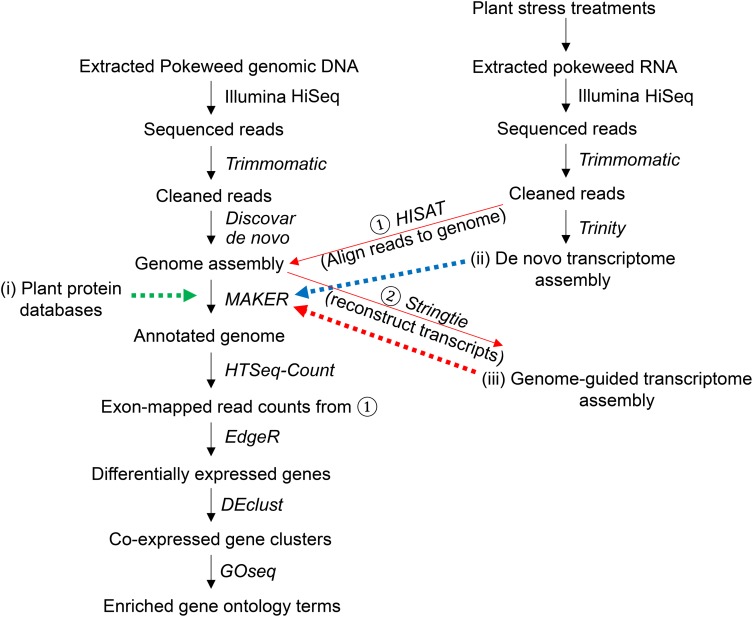
Summary of genome assembly, annotation, and differential gene expression analysis. The assembled pokeweed genome was annotated using three sources of information provided to MAKER, indicated with dashed arrows: (i) published plant protein databases, (ii) the pokeweed *de novo* transcriptome assembly, (iii) the pokeweed genome-guided transcriptome assembly, derived from aligning unassembled (‘cleaned’) reads to the genome (①) and joining exonic regions into transcripts (②). Reads from (①) that aligned to exonic regions of a gene (defined by the annotated gene models) were summed. Counts were used as input for differential gene expression analysis. The top differentially expressed genes were clustered, and functional enrichment testing was performed on each cluster.

A summary of our study is provided in Figure 1. High-quality genomic DNA was isolated from a single pokeweed plant using a CTAB-based extraction method (Healey et al., 2014) and sent to McGill University and Genome Quebec Innovation Centre (Montreal, QB, Canada) for sequencing. Shotgun sequencing was performed on one lane of an Illumina HiSeq 2500 instrument in Rapid Run mode. Paired-end reads of 250 bp were obtained from genomic DNA fragments having an average size of 400 bp; this strategy was chosen to conform with input recommendations of the downstream assembly software. Following sequencing, adapters and low-quality bases (Q < 30, averaged over four bases) were removed with Trimmomatic (v. 0.36; Bolger et al., 2014). The pokeweed genome was assembled with Discovar *De Novo* using default parameters (v. 52488; Love et al., 2016) on the Sharcnet high-performance computing cluster^1^. Raw genomic DNA sequencing reads are available at the SRA under project # PRJNA544344. The genome completion score was measured with BUSCO (v. 3; Waterhouse et al., 2018).

### Determination of Genome Size

DNA contents of somatic nuclei (pg/2C) were measured using flow cytometry. For each sample, approximately 1 cm^2^ of fresh pokeweed leaf and 2 cm^2^ of fresh *Sorghum bicolor* Pioneer 8695 (1.74 pg/2C; Johnston et al., 1999) were chopped with a razor blade in 0.7 mL of ice-cold LB01 buffer (Dolezel et al., 1989) containing 100 μg/mL propidium iodide and 50 μg/mL RNAse A. Samples were stained at room temperature for 20–25 min, then tested at low speed using a BD FACSCalibur flow cytometer (BD Biosciences, San José, CA, United States). The FL2 detector (585/42 nm) was used to measure fluorescence, with integrated fluorescence as the parameter of interest. 2C DNA contents were calculated based on the relative fluorescence of the pokeweed and *Sorghum* G0/G1 nuclei and the known DNA content of *Sorghum*. Means, coefficients of variation, and nuclei numbers for the nuclei fluorescence peaks were measured using flowPloidy (v. 1.7.0; Smith et al., 2018). Nine pokeweed plants were tested in total: four as individuals and five as bulk samples of two or three plants.

### Stress Treatments, mRNA Sequencing, and Transcriptome Assembly

Total RNA was extracted from four-leaf pokeweed plants subjected to the following treatments: sprayed with 5 mM JA or SA (solubilized in 0.5% ET), watered every 3 days for a 7-day period with 10% PEG, or wounded with forceps (WND). Plants sprayed with 0.5% ET or WT served as controls for JA/SA and PEG/WND, respectively. Leaf tissue was flash-frozen in liquid nitrogen 24 h following treatment for JA, SA, ET, and WND samples, and 3 days after the final treatment for PEG and WT samples. RNA-Seq libraries were constructed with the TruSeq Stranded mRNA Library Preparation Kit (RS-122-2101, Illumina). For each condition, four biological replicate libraries were prepared from equal amounts of RNA pooled from three independent plants (i.e., 24 libraries derived from 72 total plants). Strand-specific, paired-end reads of 125 bp were sequenced on two lanes of an Illumina HiSeq 2500 instrument by The Centre for Applied Genomics (The Hospital for Sick Children, Toronto, ON, Canada). Raw mRNA sequencing reads are available at the SRA under project # PRJNA309999.

In addition to the data generated in the present study, mRNA transcriptome assembly incorporated two publicly available *P. americana* RNA-Seq datasets from the SRA: Accessions SRX2774676 and ERX2099309, which were derived from whole plants (root, stem, leaf, and flower tissue), as well as high-coverage RNA-Seq datasets (*n* = 3 biological replicates) from our previous study of the pokeweed leaf mRNA transcriptome (Neller et al., 2016). All datasets (32 in total) were processed with Trimmomatic as described above. Two transcriptome assembly strategies were employed. For *de novo* assembly, reads were combined into a single reference transcriptome using Trinity (v. 2.5.1; Grabherr et al., 2011; Haas et al., 2013). For genome-guided assembly, RNA-Seq libraries were independently aligned to the assembled genome with HISAT (v. 2.1.0; Kim et al., 2015) and transcript reconstruction was performed with Stringtie (v. 1.3.4b; Pertea et al., 2015). The independent assemblies were combined into a single, non-redundant reference transcriptome using the Stringtie ‘merge’ function.

### Genome Annotation

Only contigs of minimum length 10 Kb were annotated, as these were most likely to contain full-length protein-coding genes (Campbell et al., 2014a). Prior to annotation, a pokeweed-specific repeat library was prepared with RepeatModeler (v. 1.0.9)^2^. Repeat-masking and gene prediction were performed using the MAKER pipeline (v. 2.31.8; Campbell et al., 2014a,b). Both pokeweed-specific and simple repeats were masked with RepeatMasker (v. 4.0.7)^3^. RepeatRunner was used to mask divergent protein-coding portions of retro-elements and retro-viruses not identified by RepeatMasker (Smith et al., 2007).

Gene prediction followed the protocol outlined in Campbell et al. (2014a). The Trinity (*de novo*) and Stringtie (genome-guided) transcriptomes described above were provided as transcript evidence. The proteomes of published Caryophyllales species (sugar beet, spinach, quinoa, amaranth) were used as homologous protein evidence (Dohm et al., 2014; Clouse et al., 2016; Jarvis et al., 2017; Xu et al., 2017). The quinoa and amaranth proteomes were obtained from Phytozome^4^ (v. 1.0 and v. 2.1, respectively), spinach from SpinachBase^5^ (v. 1), and sugar beet from The *Beta vulgaris* Resource (Refbeet-1.1)^6^. Protein evidence was further supplemented with the plant subset of the SwissProt database (obtained October 2017; Pundir et al., 2017). Three rounds of iterative gene prediction were performed with MAKER. In Round 1, predictions were inferred directly from transcript and protein evidence (est2genome, protein2genome = 1). Top-scoring gene models from Round 1 (AED ≤ 0.25, amino acids ≥ 50) were used to train the *ab initio* gene predictors SNAP (Korf, 2004) and AUGUSTUS (v. 3.2.3; Stanke et al., 2004). In Round 2, MAKER was re-run with *ab initio* gene predictors turned on, and top-scoring models were used to re-train predictors as per above. A final Round 3 was run with the twice-trained gene predictors.

Following gene prediction, an Interproscan (v. 5.28-67.0; Zdobnov and Apweiler, 2001) search was performed to identify protein-coding (Pfam) domains and obtain associated GO terms. GO annotations were supplemented with non-redundant GO terms from the SwissProt database for the best hit as per BLAST-P analysis. Each round of genome annotation was inspected visually with JBrowse (v. 1.12.3 Buels et al., 2016). In Rounds 2 and 3, we observed spurious fusion of protein-coding gene models with putative pseudogenes; this could not be resolved despite several attempts of re-training and post-annotation processing. Therefore, the final gene set was conservatively chosen to comprise only the evidence-based annotations from Round 1. The completion score of the annotated gene set was measured with BUSCO as described above, as well as relative to CoreGFs of the ‘green plants’ subset from PLAZA v. 2.5 (Veeckman et al., 2016).

### Validation of PAP Isoform Gene Models

cDNAs were generated by reverse transcribing 0.5 μg of total pokeweed RNA with SuperScript III reverse transcriptase (25 units; Thermo Fisher) and isoform-specific primers. To validate PAP isoform gene models at both the genomic and mRNA levels, PCR was performed with isoform-specific forward and reverse primers using either pokeweed gDNA or cDNA as the starting template. All PCR amplifications were conducted using Q5 High-Fidelity DNA polymerase (1 unit; New England Biolabs) and 0.5 μM of each primer. PCR products were gel-purified and used as templates for a second round of PCR, this time with forward and reverse primers that contained additional sequences for cloning. Amplicons from the second round of PCR were gel-purified and cloned into the multiple cloning site of pHSG299 vector using the one-step SLIC (sequence- and ligation-independent cloning) method (Jeong et al., 2012). All constructs were sequenced and compared with computationally derived gene and mRNA models. Primers used in this study are listed in Supplementary Table 1.

### Orthogroup Analysis

The longest isoform per gene was obtained for each Caryophyllales proteome noted above, and orthogroup assignment was performed with OrthoFinder (v. 2.2.6; Emms and Kelly, 2015). Functional enrichment analysis of genes from pokeweed-specific orthogroups was conducted with the GOseq package (Young et al., 2010) in the statistical program R (R Foundation for Statistical Computing, 2016). Pokeweed-specific genes were tested for enriched GO terms relative to all pokeweed genes.

### Differential Gene Expression Analysis

HTSeq (v. 0.8.0; Anders et al., 2015) was used to sum exon-level counts per gene for each independent RNA-Seq library aligned to the pokeweed genome. Gene counts were provided as input for differential expression testing in the Bioconductor package EdgeR (v. 3.7; Robinson et al., 2010). All pairwise tests were performed with four biological replicates per treatment, and genes with FDR < 0.05 in at least one comparison were considered differentially expressed. Treatment-responsive genes were identified based on the relevant tests: ET vs. JA or SA, and WT vs. PEG or WND. Top DEGs were defined as those with FDR < 0.001 and FC > 4 in at least one pairwise comparison. These genes were clustered with DEclust (v. 1.0.1; Aoto et al., 2017) based on a multi-conditional expression profile of EdgeR results and normalized abundance in TPM. GO enrichment analysis was performed on the genes in each cluster as described above.

### Quantitative RT-PCR (qRT-PCR) Validation of PAP Isoform mRNA Levels

cDNAs were generated by reverse transcribing 0.5 μg of total pokeweed RNA with SuperScript III reverse transcriptase (25 units; Thermo Fisher) and gene-specific primers (Supplementary Table 1) according to the manufacturer’s instructions. Elongation factor-1-gamma (EF1G) and the cell wall protein BIIDXI served as internal controls as these transcripts were stably expressed under our stress treatments according to our RNA-Seq differential expression analysis. Following cDNA synthesis, 5 μL of the reverse transcription (RT) reaction product was combined with 0.3 μM forward primer, 0.3 μM reverse primer, and 33 μL of 2X SYBR Green qPCR Master Mix (Bimake), to a final volume of 66 μL. Each reaction was split into three technical replicates. qRT-PCRs were conducted in a QIAGEN RotorGene Q thermocycler with the following settings: hold at 50°C for 20 s, initial denaturation and hot-start DNA polymerase activation for 10 min, followed by 40 cycles alternating between denaturation (95°C; 15 s) and combined annealing/extension (68°C, 45 s). mRNA levels were quantified using the ΔΔCt method (Livak and Schmittgen, 2001). A melting curve analysis was performed to confirm the presence of a single PCR product after each reaction. At least three biological replicates per treatment were conducted for each transcript.

### Generation of Promoter-GUS Reporter Constructs

The 1262 bp region upstream of the PAP-I TSS was considered the proximal PAP-I promoter. This ∼1.3 Kb promoter, along with the 5′ UTR and 1.6 Kb intron, were PCR-amplified from pokeweed genomic DNA (500 ng) with the primers PAP-I-prom-SLIC-FOR and PAP-I-prom-SLIC-REV (0.5 μM each). Amplicons were gel-purified and cloned by one-step SLIC (Jeong et al., 2012) into the multiple cloning site of pHSG299. All PCR amplifications were conducted using Q5 High-Fidelity DNA polymerase (1 unit; New England Biolabs) using the manufacturer’s instructions. The pHSG299 plasmid containing the PAP-I promoter and intron then served as the PCR template for all downstream PCRs of PAP-I promoter fragments. PAP-I promoter fragments containing the 1.6 Kb intron (1262-int and 102-int) were generated through PCR by pairing the same reverse primer (5-UTR-SLIC-REV) with different forward primers (P1-FOR or P7-FOR). Primer sequences are listed in Supplementary Table 1.

To produce intronless versions of promoter constructs, a reverse primer (5-UTR-no-int-REV) was designed to connect the two portions of the PAP-I 5′ UTR that were originally interrupted by the intron. This reverse primer was paired with different forward primers (P1-FOR to P7-FOR) to produce 5′ promoter truncations (1262, 1124, 711, 584, 432, 296, and 102). PCR products were gel-purified and used as templates for a second round of PCR to attach sequences needed for SLIC cloning.

Amplified fragments were cloned in place of the CaMV 35S promoter in pCambia 0305.2 using one-step SLIC (Jeong et al., 2012) and transformed into *Escherichia coli* DH5α. For constructs used to investigate the effect of the PAP-I leader intron on gene expression, the castor bean catalase intron in the GUS reporter gene was removed to determine the influence of the PAP-I intron alone. Minus catalase intron constructs were made by excluding the intron through PCR (Primer pairs: pCambia-1-no-cat-FOR and pCambia-1-no-cat-REV; pCambia-2-no-cat-FOR and pCambia-2-no-cat-REV) and reassembling the two fragments through Gibson assembly (Cat# E2611S; New England Biolabs). Positive *E. coli* transformants were screened through colony PCR, and all reporter gene constructs were confirmed by sequencing.

Constructs were transformed into *Rhizobium radiobacter* (syn. Agrobacterium) AGL1 strain by electroporation as previously described (Wise et al., 2006). Electrocompetent cells (20 μL) mixed with plasmid DNA (50 ng) were electroporated (2.5 kV, 25 μF capacitance, and 400 Ω resistance) and allowed to recover in 2 mL of non-selective YEP medium for 2 h. After recovery, 100 μL of cells were plated on selective YEP agar (50 μg/mL carbenicillin, 50 μg/mL kanamycin) and incubated at 28°C to allow colonies to form.

### Measurement of Reporter Activity (GUS Histochemical and Fluorometric Assay)

Agrobacterium cultures harboring promoter-reporter gene constructs were used to agroinfiltrate leaves of four-leaf stage *Nicotiana tabacum* plants as previously described (Zhao et al., 2017). Cultures were grown in selective YEP liquid medium (50 μg/mL carbenicillin, 50 μg/mL kanamycin) until late log phase (OD_600_ = 0.7 – 1.0). Cells were then pelleted by centrifugation, washed in agroinfiltration solution (10 mM MES-KOH, pH 5.6, 10 mM MgCl2, 200 μM acetosyringone), and resuspended in agroinfiltration solution to a final OD_600_ of 0.5. Agrobacterium cells were injected into the abaxial surface of leaves using a needleless syringe. For the JA experiment, leaves were treated with either 0 mM JA (0.5% ET; mock) or 5 mM JA 24 h after agroinfiltration. Leaf disks from inoculated plants were harvested 72 h post agroinfiltration and either used directly for histochemical assays or stored in liquid nitrogen until processing for the fluorometric assay.

GUS histochemical assays were performed according to Jefferson et al. (1987) with some modifications. Fresh leaf disks (0.5 cm diameter) from inoculated plants (minimum of three plants per construct) were vacuum-infiltrated with 5-bromo-4-chloro-3-indolyl-*b*-D-glucuronic acid, cyclohexylammonium salt (X-Gluc) solution, and incubated overnight at 37°C. After GUS staining, leaf disks were cleared of chlorophyll by washing in increasing concentrations of ET (70–100%) for 48 h.

GUS fluorometric assays were performed in black, clear-bottom 96-well plates according to Côté and Rutledge (2003), with some modifications. Frozen leaf disks (1 cm diameter; 2 disks per sample) from inoculated plants (minimum of four plants per construct) were combined with 200 mg of glass beads (1.0 mm, BioSpec) and 300 μL of GUS extraction buffer (50 mM phosphate buffer, pH 7.0, 10 mM DTT, 10 mM EDTA, 0.1% SDS, 0.1% Triton X-100, 10 mM β-mercaptoethanol), and homogenized using a 3110BX MiniBeadBeater (BioSpec) for 120 s at speed 48. Tissue debris was removed by centrifugation at 4°C and cleared plant extracts (10 μL) were mixed with 720 μL of 0.1 mM 4-methylumbelliferyl-β-D-glucuronide hydrate (4-MUG) and incubated at 37°C. Beginning at 0 min, a 10-μL aliquot was taken from each reaction every 15 min for a total of 60 min, and pipetted into a well containing 180 μL of stop buffer (0.2 M Na_2_CO_3_). Three technical replicates per time point were taken for each sample. Fluorescence values were measured at room temperature using a Synergy H4 Hybrid microplate reader (excitation: 365 nm; emission: 455 nm) and compared to a previously determined 4-methylumbelliferone (4-MU) standard curve. GUS activity was calculated from the linear slope of the fluorescence readings and normalized to the total protein concentration, which was determined using a BCA Reducing Agent Compatible Protein Assay Kit (G-Biosciences). Comparisons between mock-treated and JA-treated samples (*p* < 0.01) were performed for each promoter construct using two-tailed *t*-tests.

### Identification of Putative PAP Promoter CREs and JA-Responsive Transcription Factors

As with PAP-I, the 1.3 Kb sequence upstream of the TSS was considered the proximal promoter for each PAP isoform. Promoter sequence identity analysis was performed using Clustal Omega^7^ with default parameters. Putative plant-specific CREs were identified using PLACE^8^ and PlantPan 2.0^9^ web interfaces (Higo et al., 1999; Chow et al., 2016). To reduce false positives, only sequences with ≥90% identity to the published motifs were included. Putative TFs associated with CREs were identified in the pokeweed genome based on annotated Pfam domains, and their differential expression results were assessed for JA-responsiveness (FDR < 0.05).

## Results

### Assessment of the Pokeweed Genome Assembly

Statistics of the pokeweed genome assembly are shown in Table 1. Based on 1 Kb+ contigs, the assembly is 0.93 Gb in size and has a read coverage of 83X. The contig and scaffold N50 values are 35.2 and 42.5 Kb, respectively, where a scaffold represents the single highest coverage path through each line of the genome assembly graph. The scaffold assembly was used for all downstream analysis. The assembly had a BUSCO genome completion score of 84.3%; of a possible 1440 plant BUSCOs, 1214 were identified as complete (1142 single-copy and 72 duplicated), 82 were fragmented, and 144 were missing. For an additional metric of assembly completion, we determined the expected size of the pokeweed genome using flow cytometry (Supplementary Figure 1). Based on triplicate samples of four individual plants and two non-replicate bulk samples, the pokeweed plants used in this study had a genomic content (mean ± SEM) of 2.57 pg/2C ± 0.0019. Variability was low, with all 14 samples measuring either 2.57 or 2.58 pg/2C. Pokeweed and *Sorghum* nuclei fluorescence peaks had coefficients of variation <3.3% and included data from at least 1,000 nuclei. Given the conversion that 1 pg DNA = 980 Mb, the haploid genome size of pokeweed is estimated to be 1.26 Gb. Therefore, the present assembly represents 74% of the expected genome size.

**TABLE 1:** Statistics of the pokeweed genome assembly.

Number of input reads	330,991,096
Number of edges	1,829,078
Mean edge length (bases)	1, 111
Contig line N50 (bases)	35, 208
Number of scaffolds	847, 766
Scaffold line N50 (bases)	42, 514
Total bases in 1 Kb+ scaffolds	933,313,610
Total bases in 10 Kb+ scaffolds	821,040,380
Number of 10 Kb+ scaffolds	22, 292
Number of 1 Kb+ scaffolds	70, 834
Longest scaffold length (bases)	366, 732
Shortest scaffold length (bases)	200
Estimated read coverage	83X
BUSCO completion score	84%

### Annotation of the Pokeweed Genome

Scaffold contigs of minimum length 10 Kb were annotated with MAKER, as these were most likely to contain protein-coding genes (Campbell et al., 2014a). Accordingly, the mapping rate of pokeweed RNA-Seq reads aligned to these 10 Kb+ contigs averaged 93% over all samples. The annotation file and sequences of annotated transcripts and proteins are provided in Supplementary Data Sheets 1–3. A summary of the annotation is shown in Table 2. From 22,292 contigs, we obtained 29,773 genes (mean length = 5,072 bp) and 56,538 mRNA transcripts (mean length = 1,720 bp). Importantly, 73% of genes contained a Pfam domain, indicating that the majority are protein-coding, and 99% of genes had an AED score < 0.5 (Supplementary Figure 2), demonstrating excellent correspondence between evidence and gene models. The latter result is consistent with our evidence-only genome annotation; that is, gene models were based solely on the provided transcript and protein information rather than *ab initio* prediction.

**TABLE 2:** Statistics of the annotated pokeweed genome.

Number of genes	29,773
Number of mRNAs	56,538
Gene length	Mean: 5,072 Median: 3,452
Transcript length	Mean: 1,720 Median: 1,513
Exon length	Mean: 278 Median: 152
Intron length	Mean: 898 Median: 389
CDS length	Mean: 1,121 Median: 921
5′ UTR	Mean: 320 Median: 241
3′ UTR	Mean: 457 Median: 377
Exons per mRNA	Mean: 5 Median: 6
% of genes with Pfam domain	73%
% of genes with SwissProt hits	64%

Although we had attempted to include iterative training and *ab initio* prediction, doing so resulted in fused gene models that could not be validated by RT-PCR and PCR. Specifically, following two iterative training rounds we obtained an increase of only 72 genes (0.2%) but a substantial (30%) increase in mean gene length. Visual inspection of the annotations suggested that protein domain-containing pseudogenes had become fused with bona fide genes. This issue of spurious gene fusions upon *ab initio* incorporation persisted despite varying the gene-finding parameters, using MAKER post-processing tools, and applying the AUGUSTUS species model from the well-annotated sugar beet genome. Surprisingly, we observed that *ab initio* incorporation still resulted in high AED scores: after two iterative training rounds, 95% of genes had an AED < 0.5 (Supplementary Figure 2); this reinforces the importance of visibly inspecting gene models and associated evidence. Given that *ab initio* gene prediction resulted in only a small number of novel genes at the expense of annotation accuracy, we chose to conduct all downstream analyses with our evidence-based gene set.

### Evaluation of the Annotated Gene Set

The BUSCO completion score of the annotated pokeweed gene set was 75.8%. Since the genome assembly received a completion score of 84.3% (above), 90% of the BUSCOs identified in the genome were annotated. We also measured completion of the gene set relative to CoreGFs of the ‘green plants’ subset from PLAZA. The CoreGF reference set has a lower threshold of species conservation than BUSCO and is not limited to single-copy genes (Veeckman et al., 2016). Based on CoreGF analysis, the gene set completion score was 97.6%. Therefore, in the case of a non-model species, a low BUSCO score may be more reflective of high evolutionary divergence from reference species than incomplete genome assembly or annotation.

**FIGURE 2:**
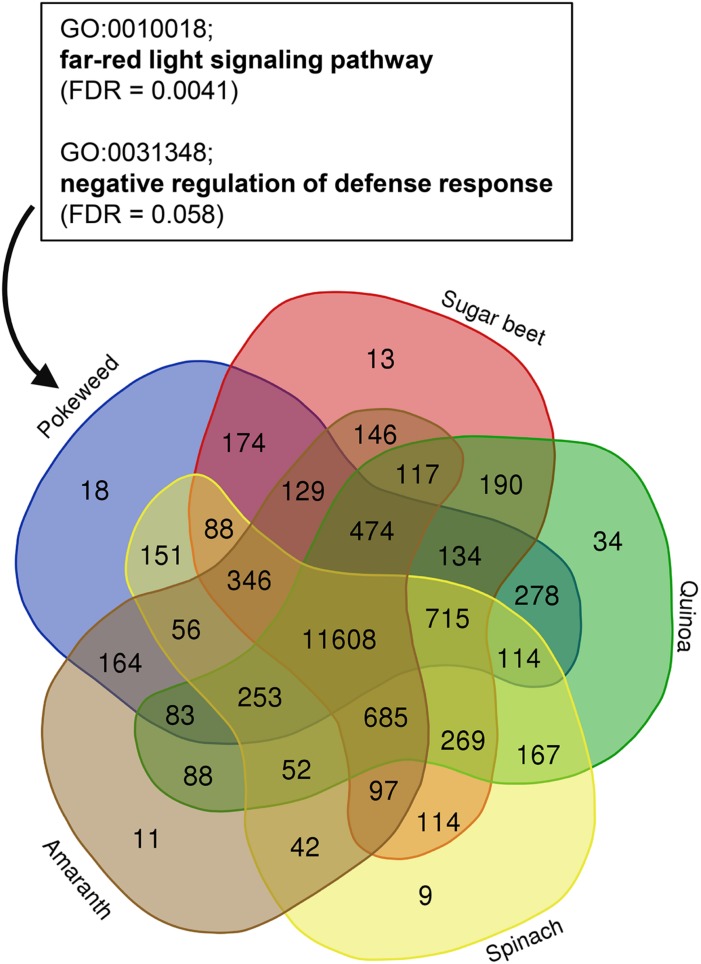
Identification of pokeweed-specific genes by orthogroup analysis of Caryophyllales species. Orthogroup assignment was performed with OrthoFinder, using the longest representative protein per gene for each species. The species distribution of orthogroups is shown. Enriched GO terms from pokeweed-specific genes (*n* = 116) are indicated in the box.

To assess how the pokeweed gene set compared to that of previously annotated Caryophyllales species, we used OrthoFinder to identify orthologous gene families (orthogroups) among pokeweed, sugar beet, quinoa, spinach, and amaranth. The species-specific distribution of orthogroups is shown in Figure 2 and Supplementary Table 2. Pokeweed genes were distributed into 14,785 orthogroups comprised of 22,901 genes. Eighteen orthogroups (116 genes) were pokeweed-specific, and these genes were enriched in the GO terms ‘far-red light signaling pathway’ (FDR = 0.0041) and ‘negative regulation of defense response’ (FDR = 0.058). The respective genes were annotated as isoforms of the TF FAR1-Related Sequence (FRS) (PHYAM_025199, PHYAM_007976, PHYAM_022646, PHYAM_016237, PHYAM_019944, PHYAM_019825) and the F-box protein Constitutive Expresser of PR genes 30 (CPR30) (PHYAM_017382, PHYAM_000825, PHYAM_000824, PHYAM_016094). For pokeweed, 77% of genes were assigned to orthogroups and 0.4% of genes were species-specific. This is in line with other Caryophyllales species, for which orthogroup-assigned genes ranged from 72% (sugar beet) to 82% (amaranth) and species-specific genes ranged from 0.1% (spinach) to 0.4% (quinoa). Taken together, these results demonstrate that the pokeweed gene set is consistent with that of well-annotated Caryophyllales species. Additionally, we have identified a subset of genes that may contribute to distinct biological relevance in pokeweed.

### Identification of RIP Genes in Pokeweed

Following genome annotation, we identified genes containing a RIP domain by performing an Interproscan search of pokeweed protein sequences against the Pfam database. This analysis revealed 10 RIP domain-containing genes, summarized in Table 3. PAP isoform annotation was made based on a BLAST-P search against the SwissProt database, retaining the best-scoring hit per protein. Three RIP domain-containing genes had 100% identity and coverage with SwissProt sequences of PAP-I, PAP-α, and PAP-S, respectively. PAP-II was also present with 99% identity and 100% coverage. One gene had 77% identity and 100% coverage with PAP-I from SwissProt, but BLAST-N against pokeweed nucleotide sequences from GenBank showed 99% identity and coverage with the partial genomic clone of PAP-S2 (Accession # AB071855.1). Furthermore, a gene annotated as PAP-S above, had 99% identity and 100% coverage with the partial genomic clone of PAP-S1 (Accession # AB071854.1). Therefore, two PAP-S isoforms exist, namely PAP-S1 and PAP-S2, which agrees with a previous finding (Honjo et al., 2002) and clarifies the single PAP-S notation in SwissProt. We also identified a gene encoding a transcript that we previously reported (c18776_g1_i1) as a potential new PAP isoform (Neller et al., 2016). The gene contains a RIP domain but only has 38% sequence identity and 96% coverage with its best hit, PAP-α, which supports this gene as a novel PAP isoform.

**TABLE 3:** Annotation of ribosome-inactivating protein genes in pokeweed.

**Gene ID**	**Scaffold ID**	**BLAST-P Top Hit**	**Annotated isoform**
PHYAM_020596	line_15290 Pos: 5980–9049	RIP1_PHYAM ID = 100%; Cov = 100%	PAP-I
PHYAM_028184	line_34803 Pos: 2685–10711	RIP2_PHYAM ID = 99%; Cov = 100%	PAP-II
PHYAM_021314	line_16387 Pos: 24092–28770	RIPS_PHYAM ID = 100%; Cov = 100%	PAP-S1
PHYAM_010467	line_4604 Pos: 41936–48641	RIP1_PHYAM ID = 77%; Cov = 100%	PAP-S2
PHYAM_022058	line_17664 Pos: 4308–7191	RIPA_PHYAM ID = 100%; Cov = 100%	PAP-α
PHYAM_012451	line_6055 Pos: 38961–39890	RIPA_PHYAM ID = 38%; Cov = 96%	Novel isoform
PHYAM_008178	line_3146 Pos: 67972–68871	RIP2_PHYDI ID = 96%; Cov = 100%	Non-transcribed pseudogene
PHYAM_008179	line_3146 Pos: 74851–75880	RIP1_PHYAM ID = 74%; Cov = 100%	Non-transcribed pseudogene
PHYAM_010465	line_4604 Pos: 18489–18926	RIP1_PHYAM ID = 79%; Cov = 46%	Transcribed pseudogene with in-frame stop codons
PHYAM_010468	line_4604 Pos: 65729–65995	RIPL2_PHYDI ID = 80%; Cov = 32%	Transcribed pseudogene with in-frame stop codons

Our analysis also led to the identification of four RIP domain-containing genes that are likely PAP pseudogenes (Table 3). Two transcribed pseudogenes were located on the same contig as PAP-S2. Their gene models were truncated relative to the associated transcript evidence, resulting in 46% and 32% coverage with the respective hits by BLAST-P. Upon closer inspection, in-frame stop codons were observed in all reading frames for both genes; this explained the shortened models, which arose from annotating the longest open reading frame per gene. Two other probable pseudogenes were identified, but their transcripts were absent in either one or both transcriptome assemblies, suggesting that the genes are transcriptionally silent. To the best of our knowledge, this is the first report of potential pseudogenes of PAP.

### Presence of a Long Leader Intron in Gene Models of PAP Isoforms

**FIGURE 3:**
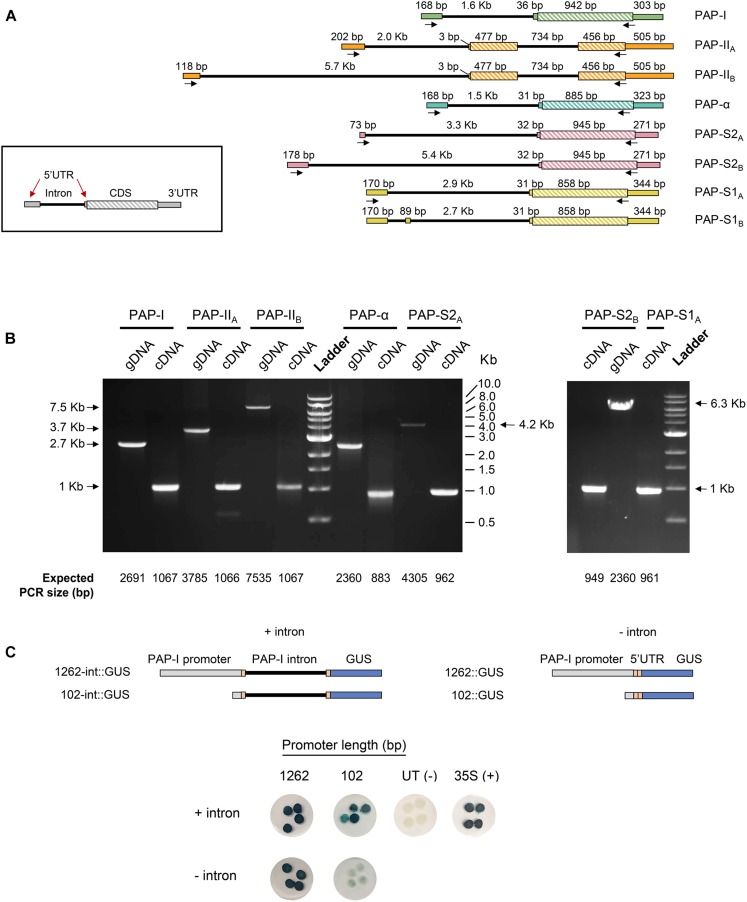
Identification of a novel intron in the 5′ UTR of PAP genes. **(A)** Gene models of PAP isoforms obtained from annotation with the MAKER pipeline. Arrows indicate primer binding sites used for gene model validation. **(B)** Validation of PAP gene models through PCR and RT-PCR from pokeweed genomic DNA (gDNA) and cDNA, respectively. All products were validated by sequencing. **(C)** Effect of the 5′ UTR intron on PAP-I gene expression. GUS reporter constructs were created with the 1262 or 102 bp PAP-I promoter, with or without the 5′ UTR intron. Constructs were agroinfiltrated into tobacco and stained for GUS. CaMV 35S = positive control; untransformed (UT) Agrobacterium = negative control. Four independent plants per construct were tested.

Gene models of protein-coding PAP isoforms are shown in Figure 3A. With exception of PAP-II, the coding sequence of all isoforms was annotated as a single exon of ∼900 bp. In contrast, the coding sequence of PAP-II comprised two exons, separated by an intron of 736 bp, which agrees with a previous report (Poyet, 1997). Interestingly, the gene models of all isoforms revealed a long intron within the 5′ UTR, ranging from 1.5 Kb (PAP-α) to 5.7 Kb (PAP-II). Based on the distribution of intron lengths in pokeweed, the PAP leader introns were longer than 83% (PAP-α) to 94% (PAP-II) of all introns. Several isoforms (PAP-II, PAP-S1, PAP-S2) were predicted to have multiple gene models that differed only in their 5′ UTRs, which suggested the use of alternative promoters in some cases (PAP-II_A_/PAP-II_B_; PAP-S2_A_/PAP-S2_B_). In addition to long leader introns, all PAP transcripts had potential upstream open reading frames in their 5′ UTRs. The majority of transcripts contained a single upstream open reading frame of four codons in length (PAP-I, PAP-α, PAP-S2_A_, PAP-S2_B_), while other transcripts contained longer ones (PAP-II_A_: 21 codons, 17 codons, 11 codons; PAP-II_B_: 8 codons; PAP-S1: 25 codons).

Where possible, gene models were validated by sequenced RT-PCR and PCR products from pokeweed total RNA and genomic DNA, respectively. As shown in Figure 3B, for each gene-specific primer pair, the PCR product size was consistent with that expected from the gene model for both cDNA and genomic DNA. The gene model of the putative novel isoform (PHYAM_012451) is not indicated in Figure 3 because its 5′ and 3′ UTRs could not be annotated; this discrepancy may be solved in the future by scaffolding with longer sequence reads. Nonetheless, we report here the validated gene models of all published PAP isoforms and the finding of a novel, conserved feature: a long intron within the 5′ UTR.

To investigate if the 5′ UTR intron affected PAP gene expression, we created GUS reporter constructs containing the PAP-I proximal promoter (1262 bp) or minimal promoter (102 bp, putative CAAT and TATA boxes only), either with or without the 5′ UTR intron. A reporter construct with the 35S CaMV promoter served as the positive control, and untransformed Agrobacterium was the negative control. Agroinfiltration of the constructs into tobacco leaves and subsequent GUS staining revealed that PAP-I promoter constructs with the intron had higher expression than those without (Figure 3C). The effect of the intron was most evident for the 102 bp promoter, where presence of the intron increased the level of GUS substantially, relative to the nearly undetectable level of staining without the intron. Based on this preliminary characterization, we hypothesize that the 5′ UTR intron, which is a conserved feature of PAP gene models, enhances PAP gene expression.

### Identification of Stress-Responsive Genes in Pokeweed

**FIGURE 4:**
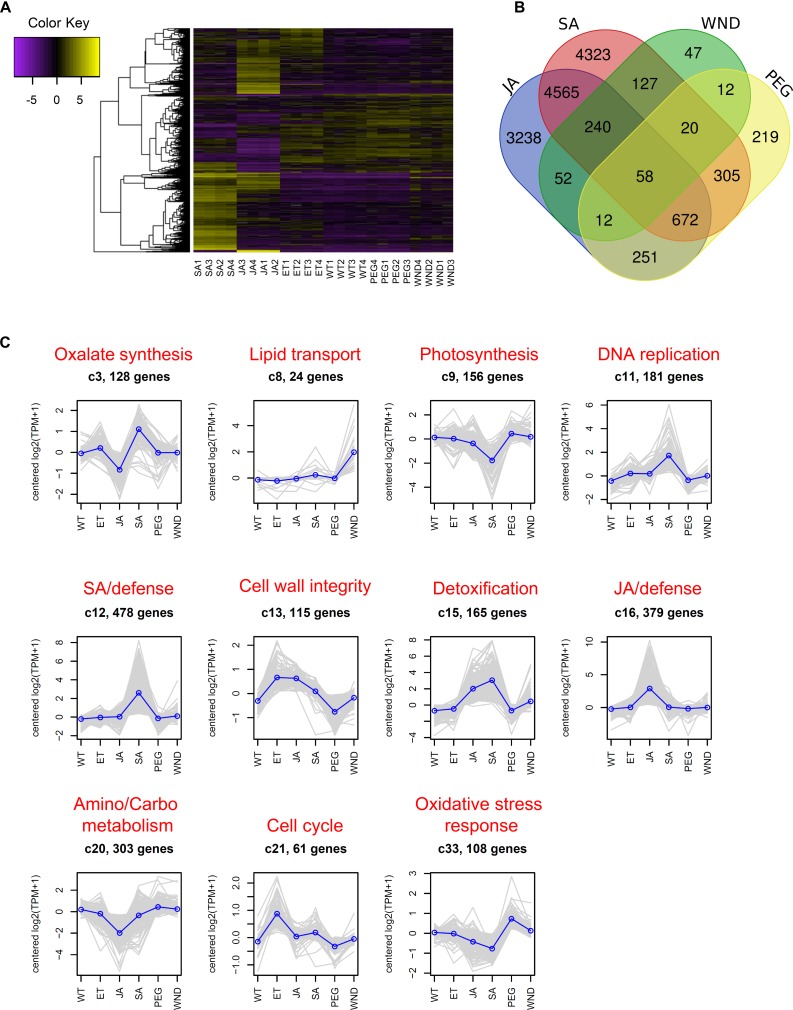
Identification of stress-responsive genes in pokeweed. **(A)** Heat map of normalized expression values [log_2_(TPM + 1), median-centered] of the top differentially expressed genes (DEGs; FDR < 0.001, FC > 4 in at least one pairwise comparison). SA, JA, ET, WND, WT, and PEG denote salicylic acid, jasmonic acid, ethanol, wounding, water, and polyethylene glycol treatments, respectively. For each condition, four RNA-Seq libraries were prepared from three independent pokeweed plants (i.e., *n* = 4 pooled biological replicates). **(B)** Treatment-specific distribution of DEGs (FDR < 0.05). **(C)** Gene clusters having significant functional enrichment. Top DEGs (from **A**) were clustered with DEclust. For each cluster, the mean expression profile was plotted as a blue line and the biological relevance of enriched GO terms (FDR < 0.05) was summarized in red font.

To gain insight into the response of pokeweed to biotic and abiotic stresses, we identified DEGs from genome-aligned RNA-Seq reads derived from several conditions. Pokeweed plants were treated with JA, SA, PEG, or WND, with ET or WT plants serving as controls for JA/SA and PEG/WND, respectively. EdgeR differential expression results and TPM-normalized expression values of all genes are provided in Supplementary Data Sheets 4, 5. Figure 4A shows a heat map of normalized expression of the top DEGs (FDR < 0.001, FC > 4 in at least one pairwise comparison). It total, 3,548 genes were differentially expressed at this threshold. The treatment-specific distribution of DEGs (FDR < 0.05) is shown in Figure 4B. The number of DEGs per treatment was as follows: SA (10,310), JA (9,088), PEG (1,549), WND (568); therefore, pokeweed was most responsive to SA and JA, which both mediate pathogen defense, and much less responsive to the abiotic stresses. Interestingly, 58 DEGs (1.6%) were common to all four treatments, including two PAP isoforms (PAP-II and PAP-α). These common DEGs were significantly enriched in the following GO terms (FDR < 0.05): ‘proline metabolic process,’ ‘ornithine metabolic process,’ and ‘putrescine biosynthetic process from arginine, using agmatinase.’ The term ‘rRNA *N*-glycosylase activity’ was also highly enriched (FDR = 0.28), suggesting that PAP contributes to more widespread stress responses in pokeweed than previously known. Furthermore, the finding that not all PAP isoforms were responsive to all treatments suggests that isoform expression is differentially regulated.

To further investigate the unique expression profiles of PAP isoforms and their potential roles in pokeweed, we identified gene clusters from the top DEGs described above. Using DEclust, which extracts statistically significant gene clusters from multi-conditional transcriptome data, 36 clusters were defined (Supplementary Figure 3 and Supplementary Data Sheet 6). Thirteen clusters revealed significant GO term enrichment (FDR < 0.05). Figure 4C shows the expression profiles of these functionally enriched clusters, and Table 4 provides the associated GO terms. Overall, each cluster had a discrete and unified biological theme, indicating successful resolution of co-expressed genes. We summarized the enriched GO terms per cluster into the following biological themes: oxalate synthesis, lipid transport, photosynthesis, DNA replication, SA-mediated signaling/defense, cell wall integrity, detoxification, JA-mediated signaling/defense, amino acid/carbohydrate metabolism, cell cycle regulation, and oxidative stress response. Two PAP isoforms (PAP-S1 and PAP-α) were present in Cluster 16, which was enriched in GO terms comprising JA-associated responses including ‘regulation of JA mediated signaling pathway,’ ‘response to wounding,’ and ‘terpenoid biosynthetic process.’ While Cluster 16 contained JA-upregulated genes, Cluster 20, which included the PAP-S2 isoform, consisted of JA down-regulated genes enriched in the terms ‘fructose-bisphosphate aldolase activity’ and ‘amino acid export.’ Overall, these results provide an indication of how pokeweed responds to diverse stresses and situate PAP within key defense pathways.

**TABLE 4:** Significantly enriched GO terms (FDR < 0.05) in pokeweed gene clusters.

**Cluster**	**Enriched terms**	**Cluster**	**Enriched terms**
3 4 8 9 11	• Oxaloacetase activity • Citramalate lyase activity • Transferase activity, transferring acyl groups other than amino-acyl groups • Lipid transport • Lipid binding • Anchored component of membrane • Chlorophyll binding • Photosystem I • Protein-chromophore linkage • Pigment binding • Photosynthesis, light harvesting in photosystem I • Plastoglobule • Response to light stimulus • Chlorophyll biosynthetic process • MCM complex • Nucleosome • DNA replication initiation • Protein heterodimerization activity • Nucleosome assembly • Cell cycle • DNA binding • THO complex • Single-stranded DNA 3′-5′ exodeoxyribonuclease activity • Embryonic root morphogenesis • Cell division • DNA replication	13	• Plant-type secondary cell wall biogenesis • Lignin catabolic process • Hydroquinone:oxygen oxidoreductase activity • Oxidoreductase activity, oxidizing metal ions • Proteinaceous extracellular matrix • Cell wall • Apoplast • Xylan *O*-acetyltransferase activity • Cellulose synthase (UDP-forming) activity • Abscission • Copper ion binding • Cellulose biosynthetic process • Rhamnogalacturonan I side chain metabolic process • Extracellular region • Xylan biosynthetic process • Hydrolase activity, hydrolyzing *O*-glycosyl compounds • Cell wall organization • Cellulose synthase complex
12	• Protein serine/threonine kinase activity • Defense response to bacterium • Respiratory chain • Plasma membrane • NADH dehydrogenase (ubiquinone) activity • ATP synthesis coupled electron transport • ADP binding • Response to salicylic acid • Signal transduction • ATP binding • Mitochondrial membrane	15 16	• Glutathione transferase activity • Serine-type endopeptidase inhibitor activity • Terpenoid biosynthetic process • Heme binding • Iron ion binding • Response to wounding • Magnesium ion binding • Metabolic process • Negative regulation of nucleic acid-templated transcription • Regulation of jasmonic acid mediated signaling pathway • Oxidoreductase activity, acting on paired donors, with incorporation or reduction of molecular oxygen
		19	• Extracellular region
		20	• Fructose-bisphosphate aldolase activity • Amino acid export
		21	• Spindle microtubule • Cell division • Chromocenter • Mitotic spindle assembly checkpoint
		33	• Membrane lipid biosynthetic process • Regulation of response to reactive oxygen species • Chloroplast membrane response to iron ion starvation

### Differential Regulation of PAP Isoform Expression

**FIGURE 5:**
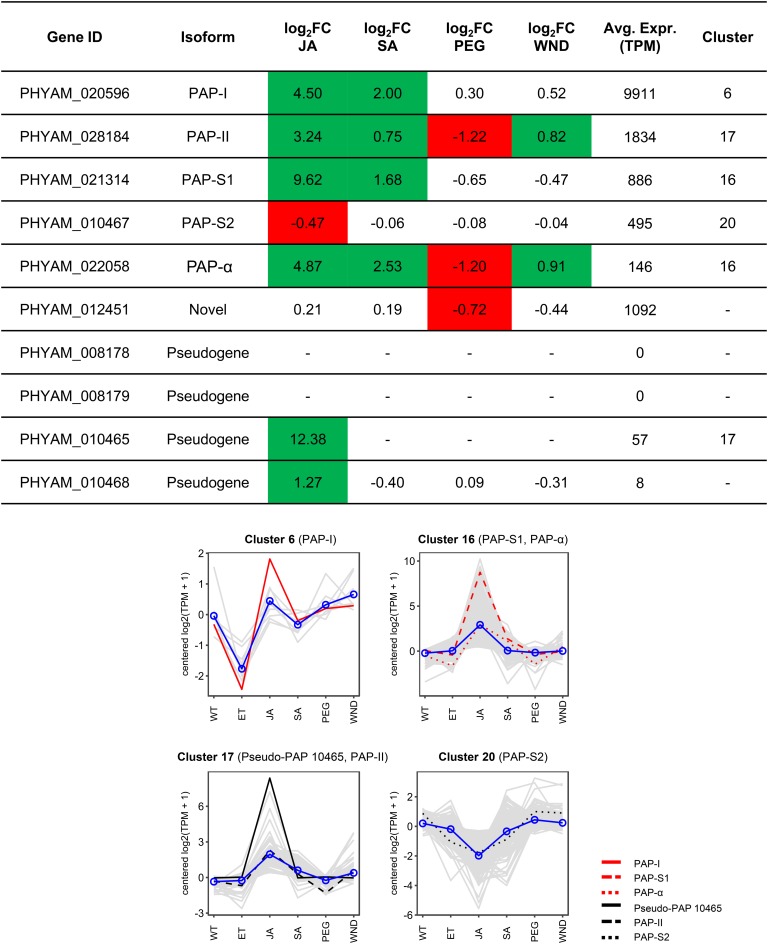
PAP gene expression profiles. **(A)** Expression changes of all PAP genes, including pseudogenes, in response to pokeweed stress treatments. Green and red indicate significant up- or down-regulation, respectively. The assigned cluster and average abundance of each isoform are also shown. **(B)** Expression profiles of PAP-containing clusters. The profile of the relevant PAP isoform (red or black line) is indicated, as well as the mean expression profile of all genes in the cluster (blue line).

The individual expression profiles of PAP genes, including pseudogenes, are provided in Figure 5A. PAP gene expression changes were also validated through qRT-PCR for all four stress treatments (*R*^2^ = 0.8807; Supplementary Figure 4). In addition to showing differences in stress-induced expression change, the isoforms varied greatly in abundance. Among protein-coding isoforms, average abundance in TPM across all samples ranged from 146 (PAP-α) to 9911 (PAP-I). PAP-I was the 24th most expressed gene in the plant under normal conditions (WT) and third most expressed upon JA treatment. As expected, only two of the four PAP pseudogenes showed quantitative evidence of transcriptional expression, and their abundances (TPM = 57 and 8) were much less than those of protein-coding isoforms (average TPM = 2,394). Both transcribed pseudogenes were JA-responsive, one of which (PHYAM_010465) was assigned to Cluster 17 with PAP-II (Figure 5B). Given that pseudogenes can regulate post-transcriptional expression of functional parental genes, our finding that a PAP pseudogene is co-expressed with a PAP isoform may indicate a novel mechanism by which PAP gene expression is controlled.

PAP-I and PAP-II, the two most abundant isoforms, were assigned to clusters that lacked significant functional enrichment (Figure 5B). PAP-I was responsive to both JA and SA. It was assigned to Cluster 6, which included only eight other genes, six of which had annotated homologs: two TFs from the HD-ZIP homeobox family (HAT5 and ATHB-7), pathogenesis-related protein STH-21, and the enzymes galactinol synthase 2, strigolactone esterase DAD2, and 1,4-dihydroxy-2-naphthoyl-CoA thioesterase. PAP-II, like PAP-α, was responsive to all stresses; however, the two isoforms were assigned to different clusters (17 and 16, respectively). The mean expression profiles of both clusters were similar, exhibiting a prominent peak with JA treatment. Accordingly, Cluster 17 was enriched in the terms ‘response to wounding’ (FDR = 0.10) and ‘jasmonic acid biosynthetic process’ (FDR = 0.23). Assignment of PAP-II and PAP-α to different clusters likely reflects differences in the intensity of their responses to JA (log_2_FC = 3.24 and 4.87, respectively) and SA (log_2_FC = 0.75 and 2.53, respectively). The putative novel isoform (PHYAM_012451) was most unlike the others. It responded only to PEG, showing a small but significant decrease in expression (log_2_FC = −0.72). Because this isoform was not among the top DEGs, it was not included in the clustering analysis; future work will investigate treatments that induce its expression. Taken together, these results demonstrate that PAP isoforms have distinct expression profiles, suggesting that they contribute to unique functions in pokeweed.

### JA-Responsiveness of the PAP-I Promoter

**FIGURE 6:**
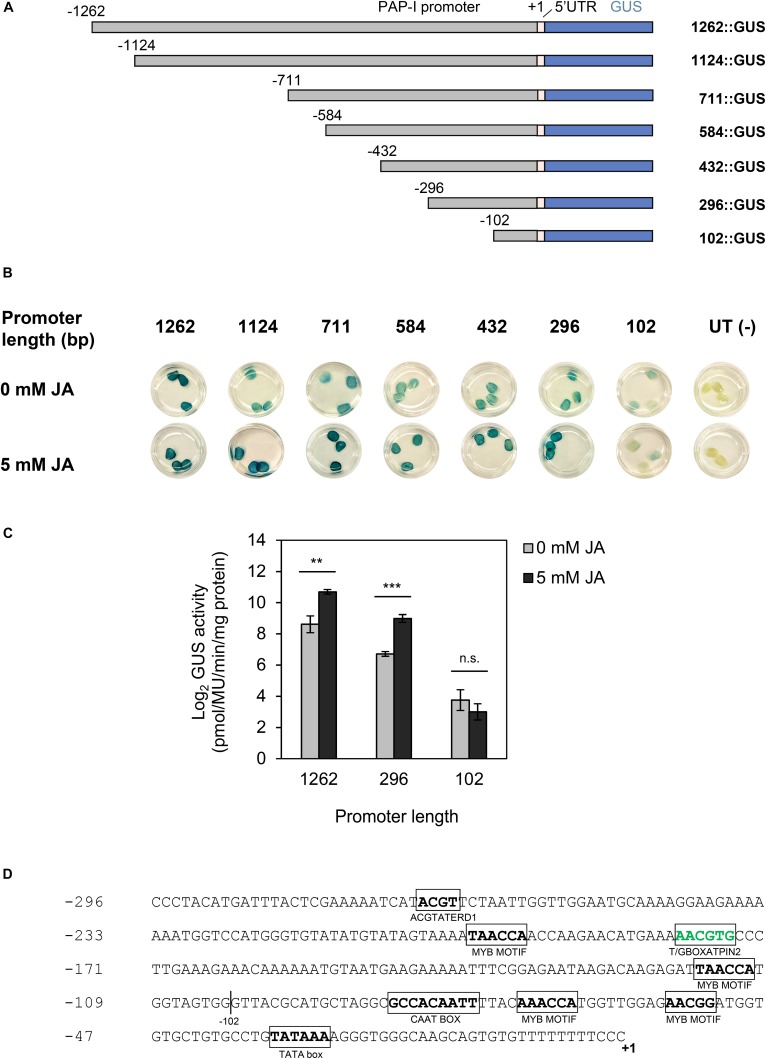
Response of the PAP-I promoter to JA. **(A)** Schematic illustration of PAP-I promoter:GUS constructs. The PAP-I promoter was serially truncated from the 5′ end. +1 denotes the transcription start site (TSS). **(B)** GUS histochemical assay in tobacco leaves agroinfiltrated with PAP-I promoter:GUS constructs and treated with either 0 mM (mock) or 5 mM JA. Untransformed (UT) Agrobacterium = negative control. Three independent plants per construct were tested. **(C)** GUS fluorometric assay in tobacco leaves agroinfiltrated with PAP-I promoter:GUS constructs and treated with either 0 mM (mock) or 5 mM JA. At least four independent plants per constructs were tested. Error bars represent the standard error of the mean (SEM). Comparisons between mock-treated and JA-treated samples were conducted for each promoter construct using two-tailed *t*-tests, *p* < 0.01. ^∗∗^*p* < 0.01; ^∗∗∗^*p* < 0.001; n.s., not significant. **(D)** Stress-related CREs in a region (–296 to +1) of the PAP-I promoter. Nucleotide position is indicated on the left, relative to the validated TSS (+1). Sequences of stress-related CREs, along with the CAAT and TATA boxes, are bolded and boxed. Green font indicates a known JA-associated element.

Since PAP-I was highly up-regulated with JA (log_2_FC = 4.5), we hypothesized that the PAP-I promoter contained CREs to mediate this response. PAP-I promoter fragments (ranging from 1262 to 102 bp) were placed upstream of the GUS reporter gene and transiently expressed in tobacco leaves through agroinfiltration (Figure 6A). Apart from those expressing 102:GUS, all plants bearing the PAP-I promoter:GUS constructs showed higher GUS activity following JA treatment, relative to controls without JA, as determined through GUS histochemical and fluorometric assays (Figures 6B,C). Therefore, our results suggest that the region upstream of the TSS (−296 to −103) is sufficient for the response of PAP-I to JA. As shown in Figure 6D, bioinformatic annotation of CREs in this region revealed an element (T/GBOXPINAT2) that binds the master JA signaling regulator MYC (Boter et al., 2004). Additionally, binding sites for TFs of the bHLH, bZIP, and MYB families were present. Specifically, MYB family members bind MYB motifs, while members of bHLH and bZIP families bind the T/GBOXATPIN2 element. Given that pokeweed homologs of several of these TFs were present in JA-associated Cluster 16 (bZIP2, bZIP11, bHLH14, bHLH25, bHLH35, MYB15, MYB44, MYB62, MYB305), the annotated CREs in this region likely contribute to JA-responsiveness of the PAP-I promoter.

### Stress-Associated CREs in the Promoters of PAP Isoforms

To gain further insight into the function of PAP isoforms, we extended our bioinformatic annotation of CREs to include the proximal promoters of all protein-coding PAP genes, including the putative alternate promoters of PAP-II and PAP-S2 (Table 5). All promoters contained TATA boxes −35 to −25 bp upstream of the putative TSS, consistent with other eukaryotic promoters. We also identified CREs associated with diverse biotic and abiotic stresses, such as T/GBOXATPIN2 (JA), W-boxes (SA), ABRE motifs (ABA), CCAAT boxes (heat stress), GARE motifs (gibberellic acid, GA), and MYB motifs (drought). Although some CREs were present in all PAP promoters (e.g., EBOXBNNAPA, GT1CONSENSUS, MYB1AT, and WBOXATNPR1), most elements differed in abundance and distribution. For instance, most ABRE motifs were absent in the PAP-α, PAP-S1, and PAP-S2_A_ promoters, while T/GBOXATPIN2 was only present in PAP-I, PAP-α, and PAP-S2_B_. Other elements were unique to a single promoter, such as ELRECOREPCRP1 (PAP-II_B_), GADOWNAT (PAP-II_A_), and WBOXNTCHN48 (PAP-II_B_). The two PAP-II promoters had only 50.5% sequence identity to each other, and the two PAP-S2 promoters had only 47.2% identity. Therefore, stimuli-dependent promoter selection in these two isoforms may lead to the transcription of distinct populations of mRNAs differing only in their 5′ UTRs. Overall, differences in the abundance and distribution of CREs in PAP promoters suggest that the isoforms have distinct roles and that control of expression can be fine-tuned by isoforms with more than one promoter.

**TABLE 5:** Putative stress-responsive *cis*-regulatory elements (CREs) in the PAP promoters.

**CRE**	**Motif**	**Response**	**Copy number in PAP promoter**							**References**
				
			**I**	**II_A_**	**II_B_**	**α**	**S1**	**S2_A_**	**S2_B_**	
ABRELATERD1	ACGTG	ABA; Dehydration	2	1	3	1	–	1	2	Simpson et al., 2003
ABREMOTIFAOSOSEM	TACGTGTC	ABA	–	1	–	–	–	–	–	Hobo et al., 1999
ABREOSRAB21	ACGTSSSC	ABA	1	–	–	–	–	–	–	Marcotte et al., 1989
ABRERATCAL	MACGYGB	ABA; Ca^2 +^	2	–	1	–	–	–	–	Kaplan et al., 2006
ACGTATERD1	ACGT	Dehydration	6	2	4	4	–	2	14	Simpson et al., 2003
ANAERO1CONSENSUS	AAACAAA	Low oxygen	1	2	1	1	–	1	2	Mohanty et al., 2005
ANAERO2CONSENSUS	AGCAGC	Low oxygen	–	–	2	–	–	–	–	Mohanty et al., 2005
BIHD1OS	TGTCA	Disease	1	2	4	6	5	4	6	Luo et al., 2005
CACGTGMOTIF	CACGTG	Defense-related	–	–	2	–	–	–	–	Gu et al., 2002
CAREOSREP1	CAACTC	GA	2	1	1	1	1	1	–	Sutoh and Yamauchi, 2003
CCAATBOX1	CCAAT	Heat stress	4	6	2	–	1	–	2	Rieping and Schöffl, 1992
DPBFCOREDCDC3	ACACNNG	ABA	–	2	1	–	2	–	1	Kim et al., 1997
DRE2COREZMRAB17	ACCGAC	ABA; Dehydration	–	2	–	–	1	1	–	Kizis and Pagès, 2002
DRECRTCOREAT	RCCGAC	Cold; Dehydration; High salt	–	3	–	–	1	1	–	Dubouzet et al., 2003
EBOXBNNAPA	CANNTG	Cold	6	12	8	8	2	6	12	Agarwal et al., 2006
ELRECOREPCRP1	TTGACC	Pathogen; SA; Wounding	–	–	2	–	–	–	–	Laloi et al., 2004
GADOWNAT	ACGTGTC	GA	–	1	–	–	–	–	–	Ogawa et al., 2003
GARE1OSREP1	TAACAGA	GA	–	–	1	1	–	–	–	Sutoh and Yamauchi, 2003
GARE2OSREP1	TAACGTA	GA	–	–	–	–	–	–	2	Sutoh and Yamauchi, 2003
GAREAT	TAACAAR	GA	2	1	1	2	2	2	–	Ogawa et al., 2003
GT1CONSENSUS	GRWAAW	Light-inducible; SA	17	16	16	12	13	14	14	Buchel et al., 1999
GT1GMSCAM4	GAAAAA	Pathogen; High salt	6	6	5	3	4	5	2	Park et al., 2004
LTRE1HVBLT49	CCGAAA	Cold	1	1	–	–	–	–	1	Alison Dunn et al., 1998
LTRECOREATCOR15	CCGAC	Cold; Dehydration	1	6	1	–	2	2	–	Kim et al., 2002
MYB1AT	CANNTG	ABA; Dehydration	5	4	2	6	6	6	3	Abe et al., 2003
MYB1LEPR	GTTAGTT	Defense-related	–	1	–	–	1	1	1	Chakravarthy et al., 2003
MYB2CONSENSUSAT	YAACKG	ABA; Dehydration	–	1	1	–	–	1	–	Abe et al., 2003
MYBCORE	CNGTTR	ABA; Dehydration	–	3	3	1	1	1	1	Abe et al., 2003
MYBGAHV	TAACAAA	GA	1	1	1	1	1	1	–	Gubler et al., 1995
MYCATERD1	CATGTG	Dehydration	–	–	1	–	–	1	1	Simpson et al., 2003
MYCATRD22	CACATG	Dehydration	–	–	1	–	–	1	1	Simpson et al., 2003
PREATPRODH	ACTCAT	Hypoosmolarity	–	2	1	2	–	–	1	Satoh et al., 2004
PYRIMIDINEBOXHVEPB1	TTTTTTCC	ABA; GA	1	–	–	–	1	–	1	Cercós et al., 1999
PYRIMIDINEBOXOSRAMY1A	CCTTTT	Sugar repression; GA	4	–	–	2	1	1	–	Mena et al., 2002
RYREPEATBNNAPA	CATGCA	ABA	–	1	–	2	5	4	–	Ezcurra et al., 1999
SURECOREATSULTR11	GAGAC	Sulfur	4	1	2	2	1	1	1	Maruyama-Nakashita et al., 2005
T/GBOXATPIN2	AACGTG	JA	2	–	–	1	–	–	1	Boter et al., 2004
WBOXATNPR1	TTGAC	SA	2	2	5	6	4	3	3	Yu et al., 2001
WBOXNTCHN48	CTGACY	Defense-related	–	–	2	–	–	–	–	Yamamoto et al., 2004
WBOXNTERF3	TGACY	Wounding	4	4	8	5	4	3	3	Nishiuchi et al., 2004

## Discussion

Here, we have presented the first *de novo* assembled draft genome of pokeweed and an annotation of protein-coding genes. We also identified clusters of co-expressed genes by integrating RNA-Seq data from several pokeweed stress treatments. We found that PAP isoforms localized to multiple clusters, with some isoforms clustering together, and functional enrichment analysis suggested distinct biological relevance of isoforms. Validation of PAP gene models led to the discovery of a long intron within the 5′ UTR. The sequence of the intron varied for each isoform, but its presence was consistent. For PAP-I, the intron enhanced gene expression of promoter reporter constructs in tobacco. Finally, we confirmed JA-responsiveness of the PAP-I promoter in tobacco and identified a region that mediates this response. This region, as well as the proximal promoters of all PAP isoforms, contained CREs associated with stress.

### Evaluation of Assembly and Annotation Metrics of the Pokeweed Genome

The pokeweed genome was sequenced exclusively as paired-end reads from a single short-insert library (83X coverage), and the resulting *de novo* assembly accounted for 74% of the expected size. We estimated the genome of pokeweed to be 1.3 Gb, in agreement with the previously reported value of 1.5 Gb obtained by Feulgen microdensitometry (Bennett, 2000). However, the assembly was highly fragmented (∼850,000 total scaffolds, with 70,834 scaffolds ≥ 1 Kb; N50 = 42.5 Kb). Available plant genomes have N50 values ranging from 10^3^ to 10^8^ bp (Veeckman et al., 2016). Nevertheless, the contiguity of the pokeweed genome assembly is comparable to other *de novo* assembled genomes derived from paired-end sequencing of short-insert libraries (Polashock et al., 2014; Van Hoeck et al., 2015). An assembly can also be highly complete but fragmented, as seen for a petunia species whose assembly accounted for 93% of the expected 1.1 Gb genome but had an N50 value of 17.9 Kb (Zhuang and Tripp, 2017). We acknowledge that more advanced sequencing would improve our genome assembly; however, the main goal of our draft genome was to identify PAP genes and their proximal promoters.

Annotation metrics indicate that our assembly sufficiently captured the protein-coding gene content of pokeweed. BUSCO completion scores for the genome assembly and gene set were 84% and 76%, respectively. Since the CoreGF score of the gene set was high (98%), and this metric is less stringent in terms of species conservation (Veeckman et al., 2016), we attribute missing BUSCOs more so to the divergence of pokeweed from model plants than incomplete assembly. Indeed, BUSCO scores are known to reflect both assembly contiguity and evolutionary history of the species under study (Simão et al., 2015). Furthermore, a similar BUSCO genome completion score (77%) was obtained for a non-model plant, a seagrass species, whose assembly metrics were similar to pokeweed: 71% assembled and N50 = 36.7 Kb (Lee et al., 2016). Pokeweed annotation metrics are also consistent with standards set by MAKER developers (Campbell et al., 2014a), who consider a genome to be well-annotated if 90% of its annotations have an AED less than 0.5 and over 50% of its proteome contains a recognizable protein domain. Furthermore, annotation of a plant genome with MAKER is expected to identify at least 20,000–40,000 genes. The pokeweed gene set meets these criteria: ∼30,000 genes, 99% of which have an AED less than 0.5, and 73% of the proteome contains a Pfam domain.

### Relevance of Pokeweed-Specific Orthogroups in Plant Defense

Pokeweed-specific orthogroups were enriched in the GO terms ‘far-red light signaling pathway’ and ‘negative regulation of defense response,’ with the involved genes annotated as isoforms of FAR1-Related Sequence and CPR30, respectively. FAR1 is a TF involved in a variety of processes relating to growth and development, including light signal transduction, circadian clock and flowering time regulation, chlorophyll biosynthesis, starch synthesis, and ABA responses (Ma and Li, 2018). FAR1, together with the light-signaling factor Far-Red Elongated Hypocotyl 3 (FHY3), regulates plant defense by integrating chlorophyll biosynthesis and SA signaling in *Arabidopsis* (Wang et al., 2016). The F-box protein CPR30 also modulates defense in *Arabidopsis* (Gou et al., 2009). Plants deficient in CPR30 showed resistance to pathogen infection and induction of defense-related gene expression. Despite the dependence of both FAR1 and CPR30 on SA in *Arabidopsis*, we did not identify any significant changes in expression of these genes in any of the pokeweed stress treatments. This may reflect the fact that our stress treatments were short-term on wild-type plants and did not simulate a mutant condition. Specifically, both *cpr30* and *fhy3 far1* plants had dwarf phenotypes, indicative of disruption to wider pathways of growth and development. Our identification of pokeweed-specific orthogroups enables future comparison with the agricultural crop plants used in our analysis, to identify defense strategies unique to pokeweed.

### Annotation of PAP Isoforms in Pokeweed

Through PAP isoform annotation, we confirmed the existence of the following previously identified isoforms: PAP-I (Irvin, 1975), PAP-II (Irvin et al., 1980), PAP-S1 (Honjo et al., 2002), PAP-S2 (Honjo et al., 2002), and PAP-α (Kataoka et al., 1992). We did not find evidence for PAP-H (Park et al., 2002), PAP-C (Barbieri et al., 1989), PAP-R (Bolognesi et al., 1990), or PAP-III (Rajamohan et al., 1999) in the scaffold assembly. However, a sequence having high identity (95%) and coverage (97%) with the cDNA clone of PAP-H was identified in the edge assembly through BLAST-N. Therefore, a gene for PAP-H likely exists in pokeweed, but it was excluded from the scaffold assembly since it was lower in coverage than an alternative path through that region. PAP-H, with 67% identity to PAP-I at the protein level, was purified from *Rhizobium rhizogenes*-transformed hairy roots of pokeweed (Park et al., 2002). It is secreted as part of the root exudates and hypothesized to contribute to the inhibition of soil-borne microbe infection. We did not find evidence for PAP-C or PAP-R, which were originally purified from pokeweed cell cultures and roots, respectively. Both PAP-C and PAP-R have N-terminal sequences that are identical to PAP-I; additionally, the three isoforms are highly similar in terms of amino acid composition, molecular weight (∼29 kDa), and pI value (∼9.5) (Barbieri et al., 1989; Bolognesi et al., 1990). Based on a lack of genomic support and high biochemical similarity, we suggest that reports of PAP-C, PAP-R, and PAP-I refer to the same isoform. Minor differences in biochemical properties could be explained by experimental variability, different post-translational modifications, or allelic diversity that cannot be resolved through *de novo* assembly. Finally, we did not identify genomic evidence in support of PAP-III, originally purified from late summer leaves. Through BLAST-P, we determined that the sequence of PAP-II, from early summer leaves, is 95% identical to the sequence of PAP-III at the protein level. Mismatches in the alignment resulted from ambiguous nucleotides (x) in the PAP-III sequence, owing to lysine methylation that enabled protein crystallization in the original report (Kurinov and Uckun, 2003). Therefore, PAP-II and PAP-III have the same amino acid sequence. It is curious that the two proteins have different levels of antiviral activity and separate as distinct peaks in ion-exchange chromatography (Rajamohan et al., 1999). We hypothesize that differences in PAP-II and PAP-III arise from post-translational modifications that affect enzymatic activity. Further support for this idea comes from the finding that a rare form of N-glycosylation exists in PAP seed isoforms, and this modification is thought to contribute to their high cytotoxicity (Islam et al., 1991; Hogg et al., 2015).

### Integration of Abiotic and Biotic Stress Responses in Pokeweed

Clustering analysis enabled the identification of genes sharing a similar expression profile. We identified 36 gene clusters, 13 of which showed significant functional enrichment. While clusters associated with SA or JA were expected since these hormones have well-established roles in plant defense, we focus here on clusters revealing potential cross-talk of key biotic and abiotic stress responses in pokeweed.

The ‘cell wall integrity’ cluster included several enzymes involved in the synthesis of cellulose and lignin, which are critical components of the cell wall. Lignin creates a physical barrier against pathogens and makes plant cells more difficult for insect herbivores to penetrate and digest (Liu et al., 2018). Multiple laccases, enzymes required for lignin polymerization, were present in this cluster. A laccase was shown to mediate broad-spectrum pathogen resistance in cotton by integrating the phenylpropanoid pathway, JA biosynthesis, and balance of JA-SA defense responses (Hu et al., 2018). In pokeweed, an increase in laccase activity during Mn treatment is thought to contribute to heavy metal tolerance by reducing the level of toxic reactive oxygen species (Gao et al., 2012). Also present in this cluster were fasciclin-like arabinogalactan proteins (FLAs). These are cell surface adhesion proteins that enable cell expansion during salt stress (Shi, 2003). Consistent with their presence, the cluster expression profile showed down-regulation with PEG, indicating sensitivity to osmotic changes. Finally, the inclusion of Defective in Induced Resistance 1 (DIR1) in this cluster, an essential signaling component of systemic acquired resistance (Maldonado et al., 2002; Champigny et al., 2013), provides further indication of integrated biotic and abiotic stress responses in pokeweed.

In the ‘lipid transport’ cluster, we identified several genes encoding non-specific lipid transfer proteins (LTPs). These are small, basic, cysteine-rich proteins that localize to the apoplast and transport various lipids. LTPs are involved in the synthesis of lipid barrier polymers, such as cuticular wax, and their expression is induced by abiotic stress (Salminen et al., 2016). LTPs also appear to have a role in JA signaling. In barley, JA biosynthesis enzymes were found to produce a covalent adduct consisting of an LTP and reactive oxylipin (Bakan et al., 2006). Furthermore, exogenous application of an LTP-JA complex to grapevine produced a higher antifungal response than either component individually (Girault et al., 2008). This gene cluster had a relatively flat expression profile apart from a spike at wounding treatment, perhaps reflecting involvement of LTPs in specific activation of the wound-response branch of JA signaling (Wasternack and Song, 2017).

The ‘oxalate synthesis’ cluster included multiple genes encoding Petal Death Protein (PDP). PDP is an isocitrate lyase in senescent flower petals that catalyzes the conversion of oxaloacetate to acetate and oxalate (Lu et al., 2005). In *Medicago truncatula*, calcium oxalate plays a role in defense against chewing insects, with a clear feeding preference observed for plants defective in calcium oxalate production (Korth, 2006). Authors also found that calcium oxalate crystals were abrasive to insects during feeding and interfered with digestion. Oxalate, as oxalic acid, is used to chelate and detoxify heavy metals in plants (Yang et al., 2005). Notably, pokeweed has an intrinsically high oxalate content that is sufficient for chelating Mn at high concentrations (Dou et al., 2009). In this gene cluster, we also identified a heavy metal-associated isoprenylated plant protein. These metallochaperones have a known role in cadmium detoxification (Tehseen et al., 2010). With pathogen resistance and heavy metal tolerance being the most cited applications of pokeweed, our results provide insight into genes that may mediate cross-talk between these important activities.

### Biological Relevance of PAP in Pokeweed

Our identification of genes co-expressed with different PAP isoforms provides a foundation for exploring their regulation in the plant and biological relevance. Two PAP-containing clusters (16 and 20) revealed significant GO term functional enrichment. Cluster 20, which contained PAP-S2 and showed down-regulation with JA, was enriched in terms relating to glycolysis and amino acid transport. The respective genes were annotated as fructose-bisphosphate aldolases (FBAs) and WAT1-related proteins. WAT1 is a vacuolar transporter that promotes indole metabolism and transport in *Arabidopsis* (Ranocha et al., 2010; Denancé et al., 2013). Indole is important in plant defense as a herbivore-induced volatile priming signal (Erb et al., 2015) and precursor for secondary metabolites (Lee et al., 2015). This cluster was also enriched in FBA genes, which function in glycolysis and gluconeogenesis. Co-enrichment of genes involved in carbohydrate and indole metabolism in this cluster suggests recruitment of the shikimate pathway, which provides a route to aromatic amino acid biosynthesis from chorismate (Parthasarathy et al., 2018). Specifically, the glycolysis intermediate phosphoenol pyruvate is used as input for the shikimate pathway, and indole is synthesized from tryptophan. FBA is also involved in the Calvin cycle of photosynthesis. Interestingly, this cluster included genes encoding pentatricopeptide repeat-containing (PPR) proteins that participate in RNA editing in the chloroplast (PCMP-H61, PCMP-H51/CRR28), an activity required for accumulation of the NADH dehydrogenase-like complex of the photosynthetic electron transport chain (Okuda et al., 2007, 2009). PPRs are a large family of sequence-specific RNA-binding proteins involved in multiple aspects of RNA metabolism. Taken together, these results demonstrate PAP-S2 co-expression with genes involved in key processes of carbohydrate and amino acid metabolism, including other proteins that interact directly with RNA.

Cluster 16, which included PAP-S1 and PAP-α, contained JA-upregulated genes and had corresponding GO enrichment in JA signaling and terpenoid biosynthesis. Like Cluster 20, this cluster also indicated PAP co-expression with genes involved in metabolism, specifically with the presence of BZIP11, a sucrose-regulated TF that controls amino acid and carbohydrate metabolism and is integrated with a wider plant growth regulatory network (Hanson et al., 2008; Ma et al., 2011). Another gene in this cluster, annotated as Enhanced Downy Mildew 2 (EDM2), is an interesting candidate that links regulation of gene expression with long introns and pathogen defense. EDM2 is a chromatin regulator that promotes normal 3′ distal polyadenylation of transcripts from genes containing intronic heterochromatin, the latter arising from methylated transposons or repeats associated with long introns (Lei et al., 2013; Duan et al., 2017). EDM2 is also required for pathogen resistance in *Arabidopsis* by regulating transcript accumulation of an NB-LRR disease resistance (R) gene (Eulgem et al., 2007).

Clusters 6 and 17, which contained PAP-I and PAP-II, respectively, did not have significant functional enrichment (FDR < 0.05). However, assignment of these isoforms to independent clusters reinforces their distinct regulation in the plant. The PAP-II cluster contained 92 genes and was enriched in the GO terms ‘wounding’ (FDR = 0.10) and ‘JA biosynthetic process’ (FDR = 0.23). The PAP-I cluster contained only nine genes, including two TFs from the HD-ZIP homeobox family that regulate plant growth and leaf development in response to abiotic stresses (Söderman et al., 1996; Aoyama et al., 2007). Other genes in the PAP-I cluster support an integration with plant growth responses. This includes DAD2, an esterase that mediates strigolactone signaling (Hamiaux et al., 2012). Strigolactone is a hormone involved in branching and symbiotic interactions with soil microbes (Marzec, 2016). Also present is a gene annotated as DHNAT1, encoding an enzyme (1,4-dihydroxy-2-naphthoyl-CoA thioesterase 1) involved in the synthesis of phylloquinone (Widhalm et al., 2012). Phylloquinone is required for Photosystem 1 stability (Wang et al., 2017) and is synthesized from chorismate, the final product of the shikimate pathway described above. These findings support the idea that PAP expression regulation is tied to the broader metabolic state of the plant. Specifically, our results indicate co-expression of PAP isoforms with genes involved in amino acid and carbohydrate metabolism, suggesting a link to wider nutrient-sensing networks. Therefore, the ribosome-inactivating activity of PAP may contribute to and/or be affected by large-scale reprograming in response to plant stress. This hypothesis is strengthened given the direct antiviral activity of PAP, which could potentially mediate a trade-off between plant growth and defense in pokeweed.

### Mechanisms of Gene Expression Regulation by Leader Introns

Investigation of PAP gene models led to the discovery of a long intron in the 5′ UTR of all PAP isoforms. The sequence of the intron was different for each isoform, but its presence was conserved. We also provided evidence to support a functional role for the intron, as the PAP-I intron enhanced reporter gene expression in tobacco. Introns can influence gene expression in both plants and animals, particularly leader introns (Laxa, 2017; Shaul, 2017). The precise mechanism of intron-mediated enhancement is not known, but hypotheses at both the transcriptional and translational levels have been proposed. Leader introns may enhance transcription by creating a favorable zone for transcription initiation; that is, a region: (i) devoid of nucleosomes, (ii) marked by activating histone modifications, or (iii) associated with a factor that recruits transcriptional machinery (Gallegos and Rose, 2015). Additionally, splicing signals in the leader intron may affect mRNA processing, export, or decay, thereby affecting translation (Gallegos and Rose, 2015; Laxa, 2017). In contrast to intron-mediated enhancement, which requires the intron to be in its native orientation and position, a leader intron may act in other ways to enhance expression. For example, the intron may function as a classical enhancer by containing CREs (Kim et al., 2006) or as an alternate promoter (Morello et al., 2002, 2006; Qi et al., 2007).

Our preliminary characterization allowed us to conclude that the PAP-I leader intron enhanced expression. Interestingly, we observed that enhancement was greater when the intron was paired with the minimal PAP-I promoter than with the proximal promoter. There may be a limit to intron-associated enhancement of gene expression, particularly for promoters that drive high expression on their own. For example, when paired with its native weak promoter, the leader intron of *Arabidopsis AtMHX* increased expression by 270-fold, compared to 3-fold with the strong CaMV 35S promoter (Akua et al., 2010). Alternatively, the enhancement provided by an intron may simply be more detectable in the presence of a weak promoter. Future work will investigate the mechanism by which the leader intron enhances PAP expression.

### Transcriptional Control of PAP Isoform Expression

The promoters of all PAP isoforms contained CREs associated with diverse abiotic and biotic stresses, suggesting that PAP is broadly implicated in plant defense. Since PAP genes were most responsive to JA in our study, we aimed to identify CREs that could mediate this response. Promoter truncation constructs of PAP-I revealed that a region close to the TSS (−296 to −103) was sufficient for JA-responsiveness. This result agrees with a previous finding that CREs closer to the TSS tend to have a greater influence on transcription (Zou et al., 2011). The −296 to −103 region of PAP-I contains binding motifs for TFs from bHLH, bZIP, and MYB families, which were found to be overrepresented in JA-responsive promoters (Hickman et al., 2017). Additionally, this region contains a T/GBOXATPIN2 element, which binds the master JA signaling regulator MYC (Boter et al., 2004). Mutation of this element abolished JA-responsiveness of genes in tomato, *Arabidopsis*, and barley (Rouster et al., 1997; Boter et al., 2004).

Analysis of JA-associated CREs in the promoters of other PAP isoforms revealed that the T/GBOXATPIN2 element was also present in PAP-α and PAP-S2_B_, but not in PAP-S1, which was most JA-responsive. The lack of this element in some promoters of JA-responsive isoforms may be compensated by the presence of W-boxes; this element binds WRKY TFs, which are primarily SA-responsive (Dong et al., 2003). However, a substantial fraction of WRKYs (at least 30%) are JA-responsive in *Arabidopsis* (Schluttenhofer et al., 2014), and we identified several that were up-regulated with JA in pokeweed (homologs of WRKY3, WRKY4, WRKY22, WRKY23, WRKY24, WRKY33, WRKY40, WRKY41, WRKY49, WRKY70, and WRKY75). Combined with the knowledge that synergistic activation of gene expression can occur in the presence of both SA and JA (Mur, 2005), we hypothesize that W-boxes in the promoters of PAP isoforms contribute to their JA-responsiveness. Although the promoter of PAP-S2 contained several W-boxes, PAP-S2 expression decreased slightly but significantly with JA and was unresponsive to other treatments. It is possible that PAP-S2 has a different temporal expression profile than the other isoforms and is more responsive outside of the 24h time-point we investigated. Consistent with this hypothesis, an RNA-Seq time-course of the JA response in *Arabidopsis* revealed diverse expression patterns over the first 16 h following treatment, including distinct early and late responses (Hickman et al., 2017). In addition to CREs associated with JA, we identified CREs associated with the hormones SA, ABA, and GA. JA and SA have well-established roles in plant defense against pathogens and insect herbivores, while ABA contributes to the resistance of abiotic stresses such as drought, salinity, cold, and heat stress (Verma et al., 2016). GA, through cross-talk with ABA pathways, helps mediate the balance between dormancy and plant maturation during stress (Verma et al., 2016). Importantly, we identified differences in the CREs of PAP promoters, suggesting that the isoforms mediate individual responses to hormones.

The draft genome of pokeweed has provided new information on how PAP expression is controlled. The presence of diverse stress-responsive CREs in the promoters of PAP genes, combined with their distinct expression profiles, provides a foundation to examine the role of PAP isoforms in pokeweed. One-fifth of land plants synthesize RIPs, and these genes are often up-regulated during stress. Our study contributes to the growing body of evidence illuminating RIPs as important components of plant response to environmental change.

## Data Availability

All datasets for this study are included in the manuscript and/or the Supplementary Files.

## Author Contributions

KN and KH designed the study. KN performed the bioinformatic analyses including genome assembly, annotation, and RNA-Seq analysis, and drafted the manuscript. CD performed all cloning, gene model and RNA-seq validations, measurement of gene expression from reporter constructs, and identification of promoter elements. AP provided advice on bioinformatic analyses and data interpretation. KH edited the manuscript. All authors read and approved the final manuscript.

## Conflict of Interest Statement

The authors declare that the research was conducted in the absence of any commercial or financial relationships that could be construed as a potential conflict of interest.

## Footnotes

1 https://www.sharcnet.ca

2 http://www.repeatmasker.org/RepeatModeler/

3 http://www.repeatmasker.org/

4 https://phytozome.jgi.doe.gov

5 http://www.spinachbase.org

6 http://bvseq.boku.ac.at

7 http://www.ebi.ac.uk/Tools/msa/clustalo/

8 http://www.dna.affrc.go.jp/htdocs/PLACE/

9 http://plantpan2.itps.ncku.edu.tw/

## Abbreviations

ABA: abscisic acid
AED: annotation edit distance
CRE: *cis*-regulatory elements
DEG: differentially expressed gene
ET: ethanol
FC: fold change
FDR: false discovery rate
GA: gibberellic acid
GO: gene ontology
GUS: β-glucuronidase
JA: jasmonic acid
PAP: pokeweed antiviral protein
PEG: polyethylene glycol
RIP: ribosome-inactivating protein
SA: salicylic acid
TF: transcription factor
TPM: transcripts per million
TSS: transcription start site
UTR: untranslated region
WND: wounded with forceps
WT: watered normally.
