# Supplementary material for: De novo Assembly of the Pokeweed Genome Provides Insight Into Pokeweed Antiviral Protein (PAP) Gene Expression
Source: Front Plant Sci. 2019 Aug 6;10:1002. doi: 10.3389/fpls.2019.01002 (PMC6691146; doi:10.3389/fpls.2019.01002)
Supplement: Supplementary file 1 [file Table_1.pdf]

Supplementary Table 1. List of primer sequences used for PCR amplification and cloning. SLIC/Gibson assembly sequences are in lowercase.

Primer names	Primer sequences (5'—3')	Purpose
pLK-1-FOR	ATTGGAAGTGGATAAGCATGCAAGCTTGGC	pHSG299 vector inverse PCR
pLK-1-REV	ATTGGATTGGAAGTACTATAGTGAGTCGTATTAGAATTCACTG	
PAP-I-prom-SLIC-FOR	ctcactatagtacttccaatccaatGACATCACTCACAGGATCAGGATTGGCTG	Cloning of PAP-I promoter and intron into pHSG299
PAP-I-prom-SLIC-REV	gcttgcatgcttatccacttccaatCTTCCCTTCCTACTAGCTAGTTCCAATACTTTCGC	
pCambia-prom-FOR	ATGGCTACTACTAAGCATTTGGCTCTTGCCATCCTTG	pCambia 0305.2 vector inverse PCR
pCambia-prom-REV	GATATTTTTGGAGTAGACAAGTGTGTCGTGCTCCAC	
P1-FOR	cacacttgtctactccaaaaatatcGACATCACTCACAGGATCAGGATTGGCTG	P1 promoter truncation (1262)
P2-FOR	cacacttgtctactccaaaaatatcGAGTGTTAGCAAATCAAGAAGGAGCAAGCAC	P2 promoter truncation (1124)
P3-FOR	cacacttgtctactccaaaaatatcAATGGGTAGTCGCCCCTTCTAGGACATTATC	P3 promoter truncation (711)
P4-FOR	cacacttgtctactccaaaaatatcACTAGACTTCAGTAGTCTGTAGTCAACCCTATTTGG	P4 promoter truncation (584)
P5-FOR	cacacttgtctactccaaaaatatcCGGAGGTCTCTCTTGTATACAGCCTCTCTATCC	P5 promoter truncation (432)
P6-FOR	cacacttgtctactccaaaaatatcCCCTACATGATTTACTCGAAAAATCATACGTTCTAATTGGTTGG	P6 promoter truncation (296)
P7-FOR	cacacttgtctactccaaaaatatcGTTACGCATGCTAGGCGCCACAATTTTACAAAC	P7 promoter truncation (102)
5-UTR-SLIC-REV	gagccaaatgcttagtagtagccatCTTCCCTTCCTACTAGCTAGTTCCAATACTTTCGC	PAP-I 5'UTR reverse primer
5-UTR-no-int-REV	CTTCCCTTCCTACTAGCTAGTTCCAATACTTTCGCCCTGTAGTTAACTCACAACTTTCTTTTTTTCTTACAACG	PAP-I 5'UTR reverse primer (intronless)
pCambia-1-no-cat-FOR	CCTCAGATCTCCAGAGCCACCGCCA	pCambia 0305.2 PCR for the removal of the catalase intron in GUS
pCambia-1-no-cat-REV	CAACCAGGCACCGACGCCGTGGAAT	
pCambia-2-no-cat-FOR	tggcggtggctctggagatctgaggAACCGACGAACTAGTCTGTACCCGA	
pCambia-2-no-cat-REV	attccacggcgtcggtgcctggttgTTCTTGATTTTCCATGCCGCCTCCTTTAG	
PAP-I-FOR	ATAACTGCATGTTCTCATAAAAAAGCCTCAGCTG	PAP-I PCR (gene model validation)
PAP-I-SLIC-FOR	ctcactatagtacttccaatccaatATAACTGCATGTTCTCATAAAAAAGCCTCAGCTG	
PAP-I-REV	GTTTATGATCAGAATCCTTCAAATAGATCACCAAG	
PAP-I-SLIC-REV	gcttgcatgcttatccacttccaatGTTTATGATCAGAATCCTTCAAATAGATCACCAAG	
PAP-IIa-FOR	CTCCGGTTATATATATGGCTATGCACTGCAG	PAP-II$_A$ PCR (gene model validation)
PAP-IIa-SLIC-FOR	ctcactatagtacttccaatccaatCTCCGGTTATATATATGGCTATGCACTGCAG	

Primer names	Primer sequences (5'—3')	Purpose
PAP-IIb-FOR	GCAGTAAATCTTAACGTACGTAGAGTCCCCTACAAAG	PAP-II$_B$ PCR (gene model validation)
PAP-IIb-SLIC-FOR	ctcactatagtacttccaatccaatGCAGTAAATCTTAACGTACGTAGAGTCCCCTACAAAG	
PAP-II-REV	GATATGATTTGAATCACTCGAATTCACCAAGG	PAP-II PCR (gene model validation)
PAP-II-SLIC REV	gcttgcatgcttatccacttccaatGATATGATTTGAATCACTCGAATTCACCAAGG	
PAP-alpha-FOR	GTTATCCATCACATTGCATGTTCTCATAAAAAGCCTC	PAP-α PCR (gene model validation)
PAP-alpha-SLIC-FOR	ctcactatagtacttccaatccaatGTTATCCATCACATTGCATGTTCTCATAAAAAGCCTC	
PAP-alpha-REV	CATCCACTCTCAGCACTATCCACTTGCTACCGTTTG	
PAP-alpha-SLIC-REV	gcttgcatgcttatccacttccaatCATCCACTCTCAGCACTATCCACTTGCTACCGTTTG	
PAP-S1-FOR	CACAGAGTTATCCATCACATTGCATGCATG	PAP-S1$_A$ PCR (gene model validation)
PAP-S1-SLIC-FOR	ctcactatagtacttccaatccaatCACAGAGTTATCCATCACATTGCATGCATG	
PAP-S1-REV	GGCATTTTGTTAAGTTGCTTGGCAAGTCCC	
PAP-S1-SLIC-REV	gcttgcatgcttatccacttccaatGGCATTTTGTTAAGTTGCTTGGCAAGTCCC	
PAP-S2a-FOR	CTGCAACGCAGAGTTCTCCATCACATCAC	PAP-S2$_A$ PCR (gene model validation)
PAP-S2a-SLIC-FOR	ctcactatagtacttccaatccaatCCAACGCAGAGTTATCCATCACATTGTATG	
PAP-S2b-FOR	CCAACGCAGAGTTATCCATCACATTGTATG	PAP-S2$_B$ PCR (gene model validation)
PAP-S2b-SLIC-FOR	ctcactatagtacttccaatccaatCCAACGCAGAGTTATCCATCACATTGTATG	
PAP-S2-REV	GTCTGACAGGTTCCATTAACGTACTTAAGGAGTGCC	PAP-S2 PCR (gene model validation)
PAP-S2-SLIC-REV	gcttgcatgcttatccacttccaatGTCTGACAGGTTCCATTAACGTACTTAAGGAGTGCC	
Pseudogene-10465-FOR	CATCACTTTGCATGTTCACATAAAAAATCCTC	PAP pseudogene (PHYAM_10465) PCR (pseudogene validation)
Pseudogene-10465-REV	GTCTACAAGACAACAAGAACCATTGGCTCATTCTC	
Pseudogene-10465-SLIC-FOR	ctcactatagtacttccaatccaatCATCACTTTGCATGTTCACATAAAAAATCCTC	
Pseudogene-10465-SLIC-REV	gcttgcatgcttatccacttccaatGTCTACAAGACAACAAGAACCATTGGCTCATTCTC	
PAP-I-full REV	GATATGATTTGAATCACTCGAATTCACCAAGG	PAP-I RT
PAP-I-1-35-FOR	ATAACTGCATGTTCTCATAAAAAAGCCTCAGC	PAP-I qPCR
PAP-I-112-83-REV	CTTCCTACTAGCTAGTTCCAATACTTTCGC	
nPAP full REV	GCTTTGAGGAGCATGTGATTATAGAATGGTG	Novel PAP RT
nPAP-117-145-FOR	CGCCACGAAGCTTCGGCTATTATACCAG	Novel PAP qPCR
nPAP-214-185-REV	CAAACCCATATTGCACATACAAGTGACACC	
PAP-II-720-690 REV	CCTTGATGCCTCATTAACCATTTGAACG	PAP-II RT
PAP-II-535-566-FOR	GAAAAGAGTTACAAAGGGATGGAATCAAAGG	PAP-II qPCR
PAP-II-644-618-REV	CGTTGCATCCTTGCCGTAGATTTTACC	

**Primer names**	**Primer sequences (5'—3')**	**Purpose**
PAP-alpha-893-864 REV	CATCCACTCTCAGCACTATCCACTTGCTACCGTTTG	PAP-α RT
PAP-alpha-20-47-FOR	CACATTGCATGTTCTCATAAAAAGCCTC	PAP-α qPCR
PAP-alpha-162-136-REV	CAAGTTGAAGGTGGTTTAAGAATGAGCC	
PAP-S1-full-REV	GGCATTTTGTTAAGTTGCTTGGCAAGTCCC	PAP-S1 RT
PAP-S1-8-37-FOR	CAGAGTTATCCATCACATTGCATGCATG	PAP-S1 qPCR
PAP-S1-91-63-REV	CCGCTTCTTTCAACACTTTCACCTGTTAC	
BDX-RT	CTTCACTCTCAGGATCCGTTC	BDX RT (reference gene)
BDX-FOR	GGAGCACACTACACCTTCGCTC	BDX qPCR (reference gene)
BDX-REV	CCTGCAACCCTGATACAATGGAAG	
EF1G-RT	CTTCAAAAGGCTCCTGGTC	EF1G RT (reference gene)
EF1G-FOR	ACCAACTTCCGTGAAGTAGCAATTAAAG	EF1G qPCR (reference gene)
EF1G-REV	CTTCCCAAATGCGTACTTGCGAG	
