# Supplementary material for: De novo Assembly of the Pokeweed Genome Provides Insight Into Pokeweed Antiviral Protein (PAP) Gene Expression
Source: Front Plant Sci. 2019 Aug 6;10:1002. doi: 10.3389/fpls.2019.01002 (PMC6691146; doi:10.3389/fpls.2019.01002)
Supplement: Supplementary file 2 [file Table_2.pdf]

**Supplementary Table 2.** Summary of orthogroup assignment in Caryophylalles species. Only species with an annotated genome were included in the analysis. Orthogroup assignment was performed with OrthoFinder, using the longest representative protein per gene for each species.

| | **Pokeweed** | **Amaranth** | **Quinoa** | **Spinach** | **Sugar beet** |
|---|---|---|---|---|---|
| Number of genes | 29773 | 23843 | 44776 | 25495 | 27421 |
| Number of genes in orthogroups | 22901 | 19572 | 36604 | 19636 | 19767 |
| Number of unassigned genes | 6872 | 4271 | 8172 | 5859 | 7654 |
| Percentage of genes in orthogroups | 76.9 | 82.1 | 81.7 | 77 | 72.1 |
| Percentage of unassigned genes | 23.1 | 17.9 | 18.3 | 23 | 27.9 |
| Number of orthogroups containing species | 14785 | 14351 | 15261 | 14766 | 15299 |
| Percentage of orthogroups containing species | 87.9 | 85.3 | 90.7 | 87.8 | 91 |
| Number of species-specific orthogroups | 18 | 11 | 34 | 9 | 13 |
| Number of genes in species-specific orthogroups | 116 | 44 | 190 | 37 | 72 |
| Percentage of genes in species-specific orthogroups | 0.4 | 0.2 | 0.4 | 0.1 | 0.3 |
