# Supplementary material for: De novo Assembly of the Pokeweed Genome Provides Insight Into Pokeweed Antiviral Protein (PAP) Gene Expression
Source: Front Plant Sci. 2019 Aug 6;10:1002. doi: 10.3389/fpls.2019.01002 (PMC6691146; doi:10.3389/fpls.2019.01002)
Supplement: Supplementary file 9 [file Data_Sheet_7.docx]

**List of captions for supplementary material**

**Data Sheet 1.** MAKER genome annotation file in gff3 format.

**Data Sheet 2.** Transcript sequences of genes annotated by MAKER.

**Data Sheet 3.** Protein sequences of genes annotated by MAKER.

**Data Sheet 4.** EdgeR results of all genes for all pairwise comparisons.

**Data Sheet 5.** Average gene expression (TPM) of all annotated genes.

**Data Sheet 6.** Gene cluster assignments for top differentially expressed genes.

**Figure 1.** **Determination of pokeweed genome size using flow cytometry.** A representative histogram is shown. The endopolyploid *Sorghum* has peaks at ~200 (2C), ~400 (4C), and ~800 (8C). Pokeweed has a 2C peak at ~300 and a small G2 peak at ~600 (<10% of total nuclei).

**Figure 2.** **Cumulative fraction plot of the annotation edit distance (AED) of gene models after each round of MAKER.** An AED of zero indicates perfect congruence between the model and associated evidence (i.e. transcript and/or protein alignments). The dashed green line illustrates

that all rounds resulted in > 90% of gene models with AED < 0.5.

**Figure 3.** **Gene expression profiles of top differentially expressed genes (DEGs).** Top DEGs (FDR < 0.001, FC > 4 in at least one pairwise comparison) were clustered with DEclust into 36 clusters. For each cluster, the mean expression profile is shown as a blue line. TPM = transcripts per million. WT, ET, JA, SA, PEG, and WND, denote water, ethanol, jasmonic acid, salicylic acid, polyethylene glycol, and wounding treatments, respectively.

**Figure 4. qRT-PCR validation of RNA-seq differential expression results for PAP genes**. Linear regression analysis between qRTPCR and RNA-seq differential expression results for PAP genes (PAP-I, novel PAP, PAP-II, PAP-α, and PAP-S1). JA, SA, WND, and PEG denote jasmonic acid, salicylic acid, wounding, polyethylene glycol treatments, respectively. R2 = 0.8807, showing high correlation between the two methods of determining transcript abundance. Results for qRT-PCR are from at least three independent biological replicates for each transcript.

**Table 1. List of primer sequences used for PCR amplification and cloning.** SLIC/Gibson assembly sequences are in lowercase.

**Table 2. Summary of orthogroup assignment in Caryophylalles species.** Only species with an annotated genome were included in the analysis. Orthogroup assignment was performed with OrthoFinder, using the longest representative protein per gene for each species.
