# Supplementary material for: De novo Assembly of the Pokeweed Genome Provides Insight Into Pokeweed Antiviral Protein (PAP) Gene Expression
Source: Front Plant Sci. 2019 Aug 6;10:1002. doi: 10.3389/fpls.2019.01002 (PMC6691146; doi:10.3389/fpls.2019.01002)
Supplement: Supplementary file 10 [file Image_1.pdf]

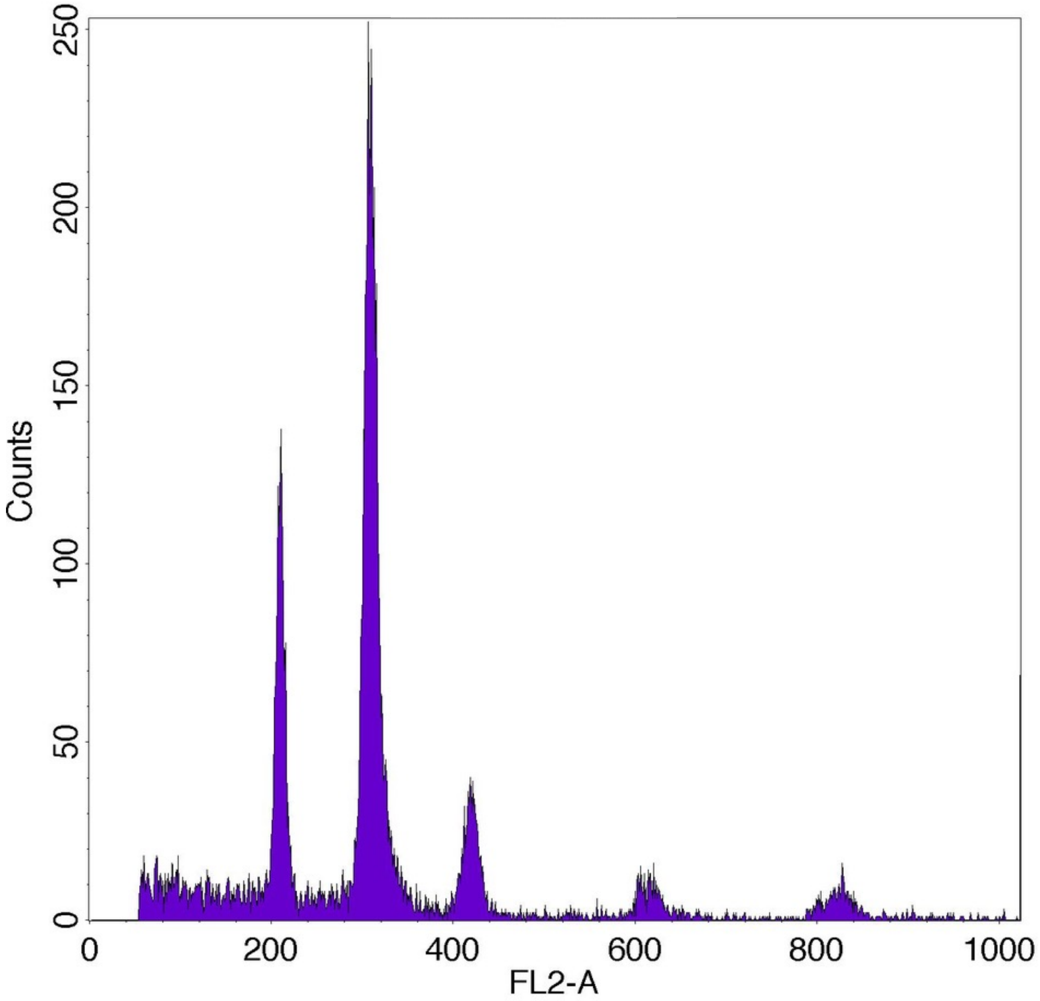

Supplementary Figure 1. Determination of pokeweed genome size using flow cytometry. A representative histogram is shown. The endopolyploid *Sorghum* has peaks at ~200 (2C), ~400 (4C), and ~800 (8C). Pokeweed has a 2C peak at ~300 and a small G2 peak at ~600 (<10% of total nuclei).
