# Supplementary material for: De novo Assembly of the Pokeweed Genome Provides Insight Into Pokeweed Antiviral Protein (PAP) Gene Expression
Source: Front Plant Sci. 2019 Aug 6;10:1002. doi: 10.3389/fpls.2019.01002 (PMC6691146; doi:10.3389/fpls.2019.01002)
Supplement: Supplementary file 11 [file Image_2.pdf]

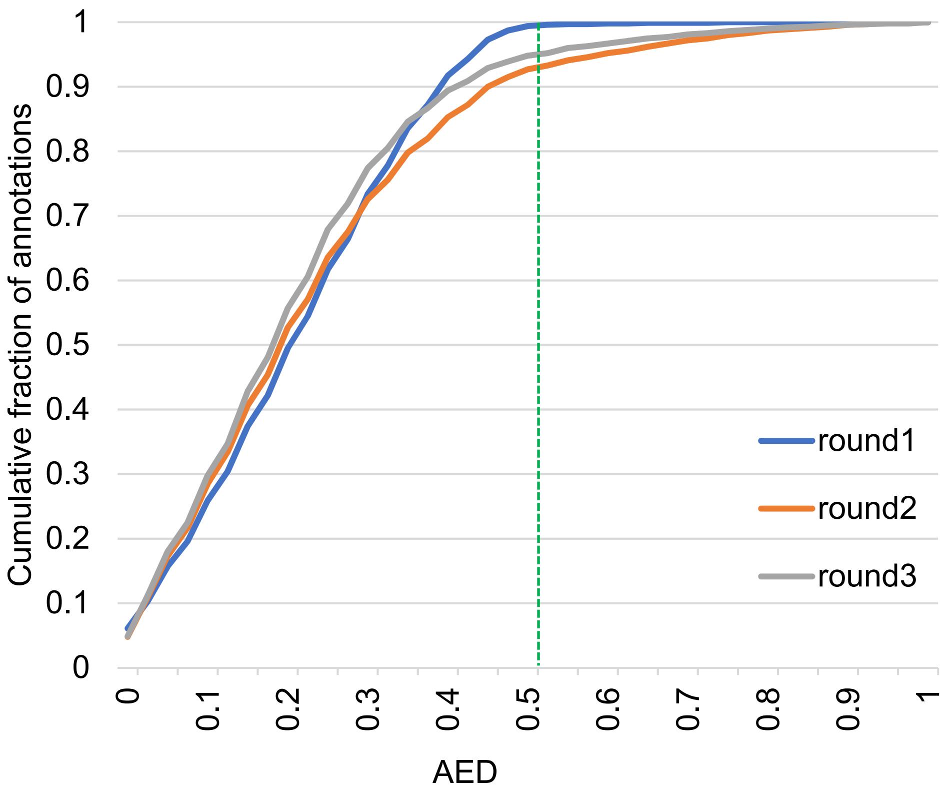

Supplementary Figure 2. Cumulative fraction plot of the annotation edit distance (AED) of gene models after each round of MAKER. An AED of zero indicates perfect congruence between the model and associated evidence (i.e. transcript and/or protein alignments). The dashed green line illustrates that all rounds resulted in > 90% of gene models with AED < 0.5.
