# Supplementary material for: De novo Assembly of the Pokeweed Genome Provides Insight Into Pokeweed Antiviral Protein (PAP) Gene Expression
Source: Front Plant Sci. 2019 Aug 6;10:1002. doi: 10.3389/fpls.2019.01002 (PMC6691146; doi:10.3389/fpls.2019.01002)
Supplement: Supplementary file 12 [file Image_3.pdf]

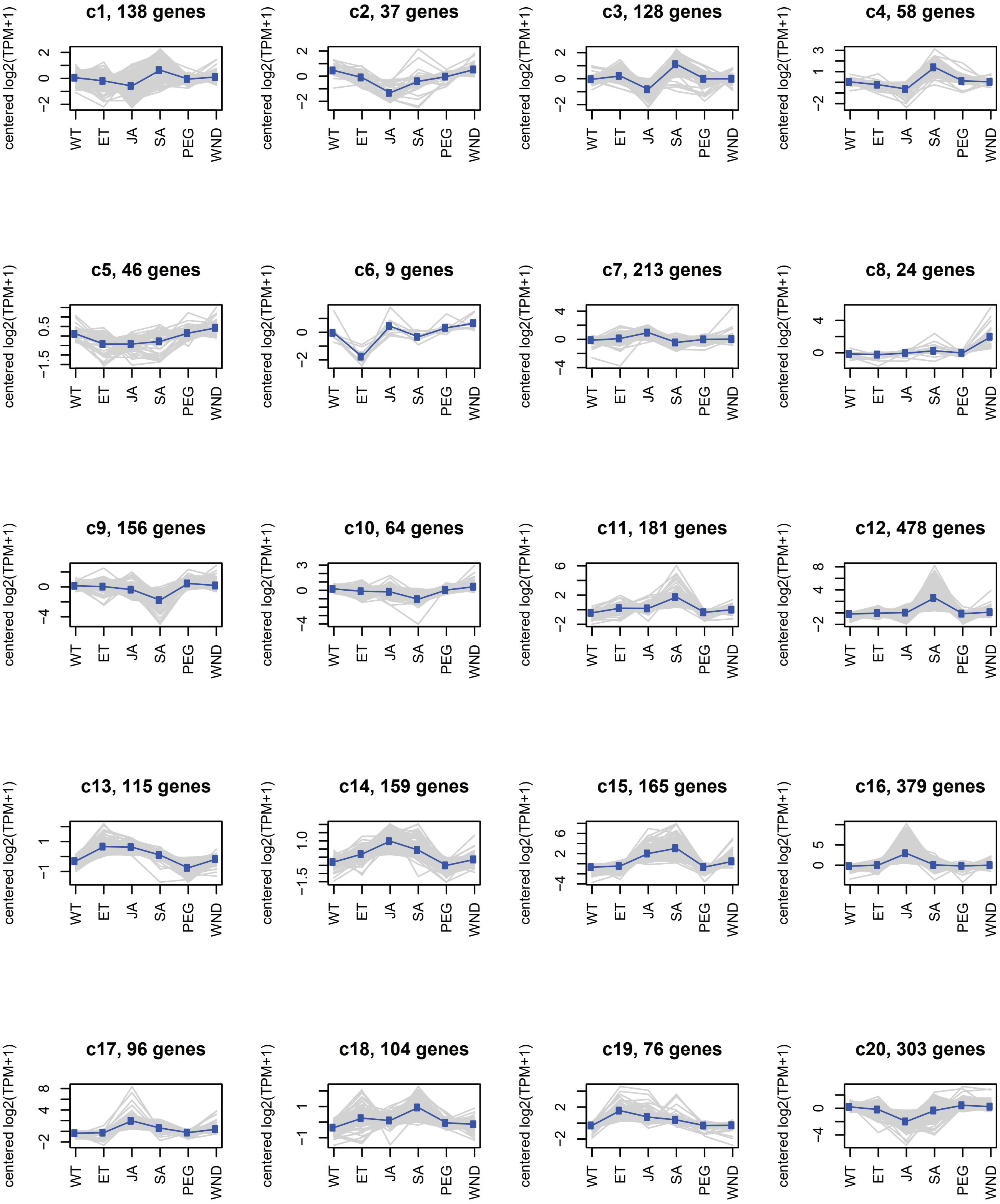
c1, 138 genes
c2, 37 genes
c3, 128 genes
c4, 58 genes
c5, 46 genes
c6, 9 genes
c7, 213 genes
c8, 24 genes
c9, 156 genes
c10, 64 genes
c11, 181 genes
c12, 478 genes
c13, 115 genes
c14, 159 genes
c15, 165 genes
c16, 379 genes
c17, 96 genes
c18, 104 genes
c19, 76 genes
c20, 303 genes
centered log2(TPM+1)
WT
ET
JA
SA
PEG
WND

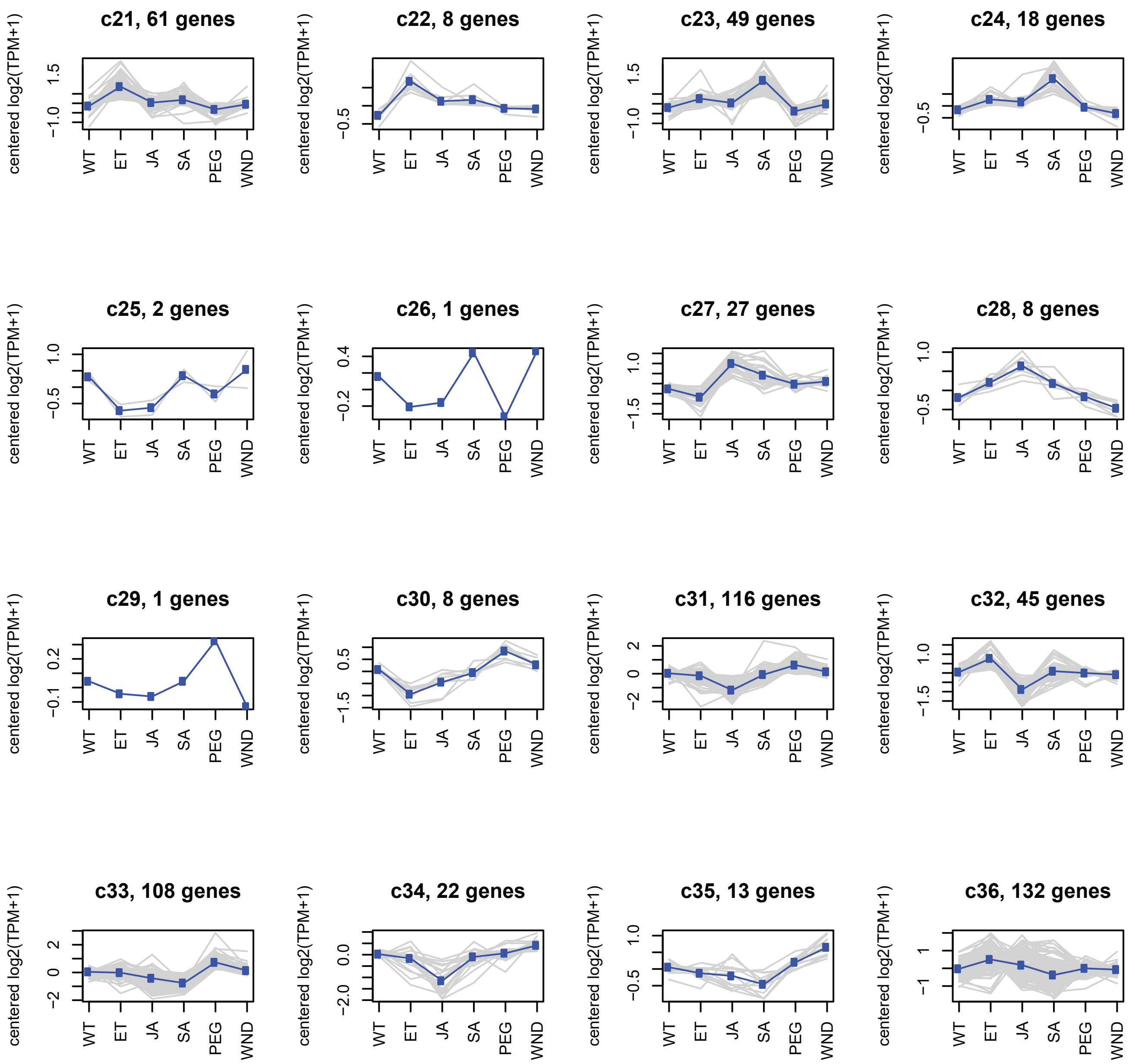

Supplementary Figure 3. Gene expression profiles of top differentially expressed genes (DEGs). Top DEGs (FDR < 0.001, FC > 4 in at least one pairwise comparison) were clustered with DEclust into 36 clusters. For each cluster, the mean expression profile is shown as a blue line. TPM = transcripts per million. WT, ET, JA, SA, PEG, and WND, denote water, ethanol, jasmonic acid, salicylic acid, polyethylene glycol, and wounding treatments, respectively.
