# Supplementary material for: De novo Assembly of the Pokeweed Genome Provides Insight Into Pokeweed Antiviral Protein (PAP) Gene Expression
Source: Front Plant Sci. 2019 Aug 6;10:1002. doi: 10.3389/fpls.2019.01002 (PMC6691146; doi:10.3389/fpls.2019.01002)
Supplement: Supplementary file 13 [file Image_4.pdf]

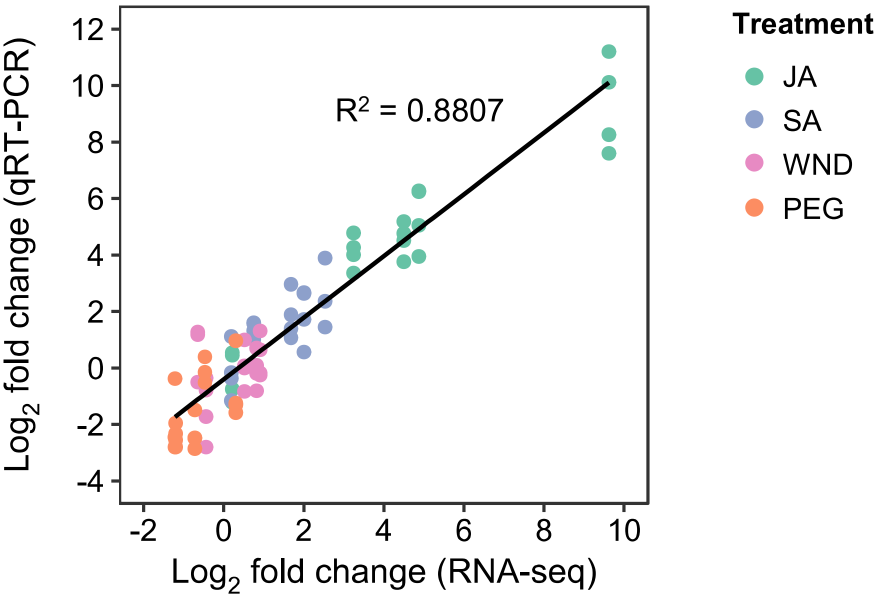

Supplementary Figure 4. qRT-PCR validation of RNA-seq differential expression results for PAP genes. Linear regression analysis between qRT-PCR and RNA-seq differential expression results for PAP genes (PAP-I, novel PAP, PAP-II, PAP-α, and PAP-S1). JA, SA, WND, and PEG denote jasmonic acid, salicylic acid, wounding, polyethylene glycol treatments, respectively. R^2 = 0.8807, showing high correlation between the two methods of determining transcript abundance. Results for qRT-PCR are from at least three independent biological replicates for each transcript.
